# The cost-effectiveness of neonatal versus prenatal screening for congenital toxoplasmosis

**DOI:** 10.1371/journal.pone.0221709

**Published:** 2019-09-18

**Authors:** Christine Binquet, Catherine Lejeune, Valérie Seror, François Peyron, Anne-Claire Bertaux, Olivier Scemama, Catherine Quantin, Sophie Béjean, Eileen Stillwaggon, Martine Wallon

**Affiliations:** 1 INSERM, CIC1432, Module Epidémiologie Clinique, Dijon, France; 2 Centre Hospitalier Universitaire Dijon-Bourgogne, Centre d’investigation Clinique, Module Épidémiologie Clinique/ Essais Cliniques, Dijon, France; 3 Aix Marseille Univ, IRD, AP-HM, SSA, VITROME, Marseille, France; 4 IHU-Méditerranée Infection, Marseille, France; 5 Hospices Civils de Lyon, Hôpital de la Croix-Rousse, Institut de Parasitologie et de Mycologie Médicale, Institut des Agents Infectieux, Lyon, France; 6 Centre Hospitalier Universitaire Dijon-Bourgogne, Délégation à la recherche Clinique et à l’Innovation, Unité de Soutien Méthodologique à la Recherche, Dijon, France; 7 Haute Autorité de Santé, Service Evaluation Economique et Santé Publique, St Denis, France; 8 Centre Hospitalier Universitaire Dijon-Bourgogne, Service de biostatistiques et d’informatique médicale, Dijon, France; 9 INSERM, UVSQ, Institut Pasteur, Université Paris-Saclay, Biostatistics, Biomathematics, Pharmacoepidemiology and Infectious Diseases (B2PHI), Paris, France; 10 Université de Bourgogne, Laboratoire d’économie de Dijon, Dijon, France; 11 CNRS, UMR 6307, Dijon, France; 12 Department of Economics, Gettysburg College, Gettysburg, Pennsylvania, United states of America; 13 INSERM, U1028, Physiologie Intégrée du Système d’Eveil, Lyon, France; 14 CNRS, UMR5292, Lyon, France; 15 Université Lyon, Centre de Recherche en Neurosciences, Lyon, France; University of British Columbia, CANADA

## Abstract

**Background:**

Congenital Toxoplasmosis (CT) can have severe consequences. France, Austria, and Slovenia have prenatal screening programs whereas some other countries are considering universal screening to reduce congenital transmission and severity of infection in children. The efficiency of such programs is debated increasingly as seroprevalence among pregnant women and incidence of congenital toxoplasmosis show a steady decrease. In addition, uncertainty remains regarding the effectiveness of pre- and postnatal treatments.

**Method:**

To identify cost-effective strategies, prenatal and neonatal screenings were compared using a decision-analytic model based on French guidelines and current knowledge of long-term evolution of the disease in treated children. Epidemiological data were extracted from the scientific literature and clinical data from the French Lyon cohort. Strategies were compared at one year of age, when infection can be definitively evaluated, and at 15 years of age, after which validated outcome data become scarce. The analysis was performed from the French Health Insurance System perspective and included direct medical costs for pregnant women and their children.

**Results:**

The 1-year Incremental Cost-Effectiveness Ratio showed that prenatal screening would require investing €14,826 to avoid one adverse event (liveborn with CT, fetal loss, neonatal death or pregnancy termination) compared to neonatal screening. Extra investment increased up to €21,472 when considering the 15-year endpoint.

**Conclusions:**

Prenatal screening is cost-effective as compared to neonatal screening in moderate prevalence areas with predominant Type II strains. In addition, prenatal screening, by providing closer follow-up of women at risk increases the number of occasions for education avoiding toxoplasmosis.

## Introduction

Toxoplasmosis is one of the most frequent zoonoses globally [1]. Human infection with *Toxoplasma gondii (T gondii)* occurs through ingestion of oocysts shed by cats that contaminate raw fruits and vegetables or water, and through accidental ingestion of tissue cysts in raw or undercooked meat [2]. The parasite persists lifelong as cysts with a strong tropism for the central nervous system, generally without causing any recognized symptoms [3]. When contracted during pregnancy, however, the infection may be transmitted to the fetus with various consequences depending on gestational age at the time of maternal infection. The later the infection occurs, the higher the risk of materno-fetal transmission, but generally with less severe injuries in the child [4,5]. Early congenital infection may lead to fetal death (around 3% of all cases) or to the birth of children with severe impairments (hydrocephalus, cognitive deficiencies, and developmental disabilities); later infection generally results in few or no signs at birth [5]. Retinochoroiditis, however, may occur any time after birth in all subjects with congenital toxoplasmosis (CT) [6]. Such ocular lesions were reported is in as many as 80% of untreated infected children [3].

To reduce the lifetime consequences of congenital infection, two preventive strategies are considered, prenatal and neonatal. The prenatal strategy combines education and serological testing of susceptible pregnant women (i.e. women not immune to toxoplasmosis), with three objectives: 1) to avoid maternal infection, 2) to recognize infection promptly, and 3) to detect and treat before birth any congenital infection. The prenatal approach relies on the hypotheses, sustained by indirect evidence, that: 1) early maternal treatment reduces the risk of mother-to-child transmission [7], and 2) congenital infection treated prenatally is associated with a lower risk of severe lesions [6]. France, Austria, and Slovenia have organized this prenatal strategy at a national level, including fully reimbursed retesting, every month, for France, and every two months, for Austria and Slovenia [5,8].

The neonatal strategy attempts only to prevent sequelae of congenital toxoplasmosis through systematic serological identification at birth and treatment of infected infants. This neonatal screening was implemented in Denmark until 2007 [9], and is carried out in the states of Massachusetts and New Hampshire in the United States, and in several states in Brazil and Colombia [8,10]. Postnatal screening reduces direct costs of screening, which could seem attractive given the larger proportion of pregnant women requiring screening due to decreasing seroprevalence and decreasing incidence of congenital *Toxoplasma* infection.

We compared the cost-effectiveness of neonatal and prenatal screening implemented in the French context. We used a decision-analytic model based on French practice and current knowledge of long-term outcomes in treated children, based on more than 20 years of French experience.

## Material and methods

We developed a two-arm decision-analytic model using TreeAge Pro Healthcare software (TreeAge Software Inc., Williamstown, MA, USA) structured to reflect implementation of legal recommendations in France [11,12] as well as French guidelines for maternal and congenital toxoplasmosis diagnosis and treatment [13]. It was validated by two independent experts (R. Piarroux of Sorbonne University, France and E. Petersen of Aarhus University, Denmark). Each expert assessed the global logic of the tree as well as its consistency with the practices (R. Piarroux for French practices and E. Petersen for Danish screening). The final structure of the tree is detailed in S1 Appendix. The target population was French pregnant women and their children. The consequences of congenital toxoplasmosis were based on the Lyon cohort (Toxo-Ly [5]) and considered outcomes until 15 years of age, as few data are available beyond that point.

### Description of the two strategies

The reference strategy matches current French prenatal screening, consisting of identification of susceptible women during the first trimester of pregnancy and their education and monthly retesting until delivery or infection. Tests are based on the quantification of *T*. *gondii* IgG and IgM, as detailed in the S2 Appendix describing the French protocol.

The alternative strategy identifies infected newborns through systematic screening at birth. Screening can be based on the detection of anti-*Toxoplasma* IgM only, or on both IgM and IgA.

It was assumed that, whether diagnosed prenatally or postnatally, all infected children are treated with pyrimethamine and sulphonamides (PS) for one year and followed until adolescence, according to the French protocol (see S2 Appendix).

To reflect real-life French practices, standard ultrasound surveillance was incorporated in our tree for both strategies. This allows the detection of fetal abnormalities due to congenital toxoplasmosis, even in the absence of a prenatal screening. In addition, we also included that parents and health care professionals have to choose between different options when ultrasound abnormalities are discovered (amniocentesis, abortion, treatment with PS).

### Epidemiological parameters

Screening/diagnostic test performances and probabilities of each clinical event were obtained from a systematic critical review of the available literature, detailed in S3 Appendix. Data from the Toxo-Lyon cohort, which includes all pregnant women monitored at the Croix-Rousse Hospital (Lyon University Hospital, France) since 1987 for primary *Toxoplasma* infection detected through prenatal screening [5,6], supplemented data from the literature (See S4 Appendix). When data were not available, we relied on the opinion of three experts i.e.: E Petersen, R Piarroux, and M Wallon for defining the central estimate of unknown probabilities as well as their possible range of variation. All probabilities and their sources are presented in Table 1.

**Table 1:** Epidemiological parameters, performance of screening tests, treatment efficacy, and clinical outcomes probabilities (Toxoscreen project).

Variable	Central Estimate (%)	Range for Sensitivity Analyses (%)	Sources
**EPIDEMIOLOGICAL PARAMETERS**			
Seroprevalence	36.7	10–50	[14–17]
Seroconversion suspicion (in women at risk)	0.24	0.05–1.0	[18–22]
Real seroconversions among suspected seroconversion	80	50–100	Expert opinion
Distribution of maternal seroconversion by trimester			[5]
1^st^ trimester	42.1	_	
2^nd^ trimester	30.6	_	
3^rd^ trimester	27.3	_	
Materno-fetal transmission^(a)^			[5]
1^st^ trimester	5.5	3.6–8.2	
2^nd^ trimester	23.1	19.0–27.9	
3^rd^ trimester	60.3	53.0–67.1	
Overall fetal abnormality occurrence during pregnancy	2	0.3–4	[23–28]
Overall fetal losses			[29,30]
1^st^ trimester	12.5	_	
2^nd^ trimester	2.5	_	
3^rd^ trimester	0.04	_	
Fetal loss in fetus with CT ^(b)^			[5]
1^st^ trimester	13.8	_	
2^nd^ trimester	6.2	_	
3^rd^ trimester	0	_	
Overall neonatal death	0.24	–	[31]
Neonatal death in newborn with CT^(b)^	0.08	0.00–0.33	[5];
Symptomatic CT in children with CT^(b)^			[5,32,33]
1^st^ trimester	37.9	34–85	
2^nd^ trimester	26.2	18–33	
3^rd^ trimester	1.8	0–17	
**EPIDEMIOLOGICAL PARAMETERS**			
Abnormal ultrasound in fetus with CT according to the trimester of maternal infection^(b)^			[5]
1^st^ trimester	24.1	10.3–43.5	
2^nd^ trimester	12.3	5.5–22.8	
3^rd^ trimester	1.2	0.1–4.3	
Death after amniocentesis	0.33	0.1–0.6	[5,34]
**PERFORMANCE OF SCREENING/DIAGNOSTIC TOOLS**			
Sensitivity of the maternal screening	95	90–100	Expert opinion
Specificity of the maternal screening	90	85–95	Expert opinion
Ultrasound examination sensitivity	61.4	34.8–78.3	[23,24,26,35–37]
PCR sensitivity	92.2	81–98	[38,39]
PCR specificity	100	93–100	[38,39]
Neonatal screening sensitivity (IgM)	61	42–87.7	[40–44]
Neonatal screening specificity (IgM)	98.5	92–99.9	[45]
Sensitivity of the neonatal pediatric examination	10	5–20	Expert opinion
Symptomatic congenital toxoplasmosis(CT) identified as such by the neonatal check-up among children with CT previously classified as asymptomatic	75	50–100	Expert opinion
Asymptomatic CT identified as such recognized by the neonatal check-up	80	50–100	Expert opinion
**COMPLIANCE WITH SCREENING**			
Women participating in the prenatal screening	80	50–100	Expert opinion [46]
Women attending prenatal screening in a system of neonatal screening	10	10–50	Expert opinion
**TREATMENT EFFICACY**			
Reduction of the risk of materno-fetal transmission by spiramycin	50	25–75	[5,40,47–51],
Reduction of the proportion of symptomatic congenital toxoplasmosis at birth by the reinforced maternal treatment (pyrimethamine + sulfadiazine)	0	0–20	Expert opinion [5,52]
Reduction of the severity of the infection resulting from child treatment	0	0–12	Expert opinion [5,6,52]
**SEQUELAE** ^(b)^			[5,6]
Initial extraocular lesions in children with a symptomatic congenital toxoplasmosis at birth	90.7	79.7–96.9	
Late extraocular lesions in children with a previous ocular lesion	12.9	5.3–24.9	
Ocular lesions occurrence during 15-year follow-up among children with previous extraocular lesions	53.1	38.7–67.5	
Ocular lesions occurrence during 15-year follow-up among children with asymptomatic CT at birth and with no delayed extraocular sign	26.3	22.2–30.7	
Extraocular lesions during the follow-up	1.8	0.2–6.3	
Recurrence of ocular lesions	33.8	26.1–42.2	
Active ocular lesions	17.4	11.4–25	
Ocular lesions recognition in children with congenital toxoplasmosis not identified previously	36.4	24.9–49.1	
Decreased visual acuity (whatever the level)	21.2	14.6–29.2	
Neurologic sequelae	8.3	1.0–27	
Sequelae in case of ocular and extra-ocular lesions	53.1	34.7–70.9	

^(a)^ Estimations are based on the TOXO-LY data between 1992 and 2008 in order to rely on a steady context of monthly screening and spiramycin treatment while maintaining a sufficient number of observations to allow weekly estimations.

^(b)^ Estimations are based on the TOXO-LY data between 1996 and 2008 in order to account for a homogeneous context of amniocentesis, PCR on amniotic fluid, and reinforced treatment by pyrimethamine and sulfadiazine.

Main baseline parameters were: 37% prevalence, maternal infection in 0.2% of women at risk, 50% reduction in risk of materno-fetal transmission with spiramycin treatment, 80% participation rate in systematic prenatal screening and, in the neonatal strategy, 10% prenatal screening on an individual basis. As there were very few data on reinforced prenatal treatment (described below, this includes pyrimethamine and sulfadiazine) efficacy as well as on the post-natal treatment on sequelae, in the base-case analysis we ascribe no efficacy to reinforced treatment in either arm, prenatal or neonatal.

### Effectiveness

The reference and alternative strategies were compared at two endpoints: 1) one year of age, when CT can be accurately confirmed or rejected and the major consequences of the disease as well as of the prenatal management have occurred; 2) 15 years, the latest age at which validated outcome data are available and the main sequelae have been diagnosed.

Two types of adverse events were considered: 1) strictly Toxoplasma-related events (STRE) and 2) global events. STRE are cases of congenital infection either recognized or not. Of note, STRE include toxoplasmosis-related fetal losses and neonatal deaths for the short-term assessment, and toxo-related sequelae, including delayed ocular lesions for the long term assessment. Global events (GE) include, in addition to the STRE, fetal loss, termination, neonatal death that may be due to other fetal diseases or may be induced by the management of toxoplasmosis during pregnancy (i.e. fetal loss caused by amniocentesis, undue termination for false positive results and/or ultrasound abnormalities, etc.) as these events may balance the benefit of treating infected women. In addition, in the model the events are clearly related or unrelated with toxoplasmosis; but in real life this is not the case: when a fetal loss occurs in the context of a seroconversion, it cannot generally be determined if this event is related or unrelated to toxoplasmosis. In addition, when different management options are possible, the model selects the option allowing the minimization of the number of adverse events considered as the endpoint. In order to avoid termination being systematically selected to avoid further fetal losses and neonatal deaths, either related or not to toxoplasmosis, it appeared of paramount importance to consider the combination of all the adverse outcomes that can occur as a whole, whatever their reasons and actual causes. A value of one was assigned to each STRE and then to each GE, and a value of zero to their absence. The strategy with the lowest expected number of adverse events was identified.

### Cost data and cost-effectiveness analysis

The cost-effectiveness analysis was performed from the perspective of the French Health Insurance System. The prenatal screening strategy was used as the reference strategy.

Direct medical costs for pregnant women and children are summarized in Table 2. They include all diagnostic procedures and treatments until age 1 or age 15 respectively, valued using French reimbursements. The French system of care coverage as well as hypotheses underlying the costs calculation is detailed in S5 Appendix.

**Table 2:** Tariffs of examinations and treatments—Toxoscreen project.

	Code/Source	Tariffs (euros)
***Pregnancy monitoring***		
Ultrasound examination (1st trimester)	JNQM001/CCAM^a^	36.35
Ultrasound fetal morphology and biometry (2^nd^ trimester)	JQQM018/CCAM^a^	100.20
Ultrasound fetal morphology and biometry (3rd trimester)	JQQM016/CCAM^a^	100.20
***Screening***		
Standard toxoplasmosis serology (IgG+IgM by 2 techniques)	B40/NABM^b^	10.80
Serologic confirmation (2 isotypes including IgG by two techniques on two samples)	B60/NABM^b^	16.20
***Toxoplasmosis confirmation***		
Amniocentesis	JPHJ002/CCAM^a^	68.58
Mouse inoculation	B300/NABM^b^	81.00
PCR	B600/NABM^b^	162.00
***Termination***	JND001/CCAM^a^	82.39
***Neonatal examinations***		
Consultation of pediatrician	CSM/NGAP^c^	46.00
Skull radiograph^d^	LAQK003/CCAM^a^	23.94
Transfontanellar ultrasound examination	AAQM002/CCAM^a^	37.80
Eye examination in newborns (until 28^th^ days after birth)	BGQP004/CCAM^a^	36.92
Computerized tomography of the head	ACQK001/CCAM^a^	25.27
***Treatments and related follow-up***		
Spiramycine (16 tb 3 MUI)	French Public Database of Drugs	11.10
Adiazine/ Sulfadiazine (20 tb 500 mg)		3.62
Malocide/ Pyriméthamine (20 tb 50 mg)		13.55
Lederfoline/folinic acid (30 tb 25 mg)–pregnancy		30.05
Fansidar^®^/ Sulfadoxine +Pyrimethamine (3 tbt)		1.98
Folinoral^®^ /acid folinic) (14 capsule-shape tbt 25 mg)–children		14.46
Blood count	B26 (1104)/NABM^b^	7.02
Proteinuria	B4 (2004)/NABM^b^	0.52
Ophthalmological follow-up	CS/NGAP^c^	30.00

^a:^ Classification Commune des Actes Médicaux;

^b:^ Nomenclature des Actes de Biologie Médicale;

^c:^ Nomenclature Générale des Actes Professionnels;

^d:^ This procedure is no longer used but were part of costs in the history of the screening program.

An Incremental Cost-Effectiveness Ratio (ICER) with prenatal screening as the reference was calculated, as:

$$\frac{\text{cost}\;\text{of}\;\text{neonatal}\;\text{screening}-\text{cost}\;\text{of}\;\text{prenatal}\;\text{screening}}{\text{effectiveness}\;\text{of}\;\text{neonatal}\;\text{screening}-\text{effectiveness}\;\text{of}\;\text{prenatal}\;\text{screening}}.$$

ICER was expressed in terms of cost per additional outcome avoided. According to international guidelines, costs and effectiveness were discounted at a 3% rate over the 15-year period (10).

### Sensitivity analyses

To assess the robustness of the model, given that the vast majority of our data relies on point estimates, deterministic multivariate sensitivity analyses were performed. Those analyses included the following parameters, listed in Table 1: treatment (spiramycin and pyrimethamine/sulfadiazine) efficacy, compliance with systematic prenatal screening, participation in individual prenatal screening in the neonatal screening strategy, and prevalence and incidence of toxoplasmosis in pregnancy. Those two latter parameters for sensitivity analysis were based on two scenarios: the first corresponded to a context of low prevalence and incidence as observed in the northern part of Europe and the USA (prevalence of 10% and incidence of 0.05% of women at risk [16,17,19,20,22]); the second considered a high prevalence and incidence context, such as in middle to southern European countries (50% prevalence and 1% incidence for women at risk [14,21]). In addition, the impact of variation in the performance of screening tests as well as in the efficacy of prenatal pyrimethamine/sulfadiazine treatments was also checked.

## Results

### Base case analysis

Total costs for the neonatal strategy at one year of age were estimated to be €78 lower than in the reference prenatal strategy (€773 per mother-child pair with the neonatal strategy vs. €851 with the prenatal strategy), as shown in Table 3. Neonatal screening was, however, less effective, associated with twice as many toxoplasmosis related adverse outcomes (STRE) (0.667 per 1,000 mother-child pairs vs. 0.334), and a 5.239 absolute difference in GE (156.033 per 1,000 vs. 161.272) (Table 3). The incremental cost-effectiveness ratio (ICER) indicated that the extra-cost of one additional avoided STRE—with the prenatal strategy compared to the neonatal screening strategy—was € 232,631. This means that the health system would have to spend € 232,631 more in the prenatal screening scenario than it would in the screening at birth scenario to avoid an additional STRE. The ICER was only €14,826 for global events (STRE, plus fetal losses, terminations of pregnancies, neonatal deaths due to other fetal diseases or induced by the management of toxoplasmosis during pregnancy).

**Table 3:** Expected number of events, costs, and cost per additional outcome avoided (Toxoscreen project).

Assessment period	Prenatal screening (a)	Neonatal screening (b)	Difference (b–a)
**Short term assessment (1 year)**	**Outcomes per 1000 women/children**		
***Strictly toxoplasmosis-related events (STRE)***			
Live-born children with toxoplasmosis (1)	0.332	0.660	0.328
Toxoplasmosis-related fetal losses and neonatal deaths (2)	0.002	0.007	0.005
Total STRE (= 1+2)	0.334	0.667	0.333
***Global events (GE)***			
Total GE (= STRE+ neonatal deaths, fetal losses unrelated to toxoplasmosis + abortion and fetal losses due to amniocentesis)	156.033	161.272	5.239
Mean cost per woman/child (€)	851	773	-78
Cost per additional outcome avoided			
STRE			232 631.4
GE			14 826.0
**Long term assessment (15 years)**	**Outcomes per 1000 women/children**		
***15 years–STRE***			
Sequelae	0.031	0.067	0.036
Total STRE (sequelae+(2))	0.033	0.075	0.042
***15 years–GE*** *(15 years STRE +* neonatal deaths, fetal losses unrelated to toxoplasmosis + abortion and fetal losses due to amniocentesis)	155.240	158.748	3.508
Mean cost (€)	826	751	-75
Cost per additional outcome avoided			
STRE			1 795 145
GE			21 472

(a) Reference strategy

After 15 years, neonatal screening was estimated to cost €75 less than prenatal screening but was associated with a higher expected number of *Toxoplasma*-related neurological and ocular sequelae (i.e. STRE: 0.075 per 1,000 *vs* 0.033), and of GE (158.748 per 1,000 *vs* 155.240). The cost per STRE avoided was €1,795,145 for STRE but only €21,472 when considering GE, which included all the adverse events that may occur during pregnancy, either related to toxoplasmosis and/or other diseases as well as to toxoplasmosis management.

### Sensitivity analysis

The deterministic one-way sensitivity analyses showed robust results for a wide range of estimates for the four parameters analyzed: incidence of maternal infection (0.05%–1%), prevalence in women of child-bearing age (10%–50%), compliance with screening (50–100%), and efficacy of treatment with spiramycin in preventing fetal infection (25–75%) (Tables 4 and 5).

**Table 4:** Sensitivity analyses: Short term assessment (Toxoscreen project).

Sensitivity analyses	Strictly *Toxoplasma*- related Events	Global Events
***Epidemiological Context***		
*Northern European country or United States (prevalence*: *10%*, *incidence*: *0*.*05%)*		
Differential cost^a^	-117	
Event differential per 1000 women screened^a^	0.1	5.0
Cost per additional outcome avoided	1,178,092	23,168
*Middle and Southern European countries (prevalence*: *50%*, *incidence*: *1%)*		
Differential costt^a^	-58	
Event differential per 1000 women screened^a^	1.1	5.91
Cost per additional outcome avoided	53,007	9,830
***Treatment Efficacy***		
*25% reduction of materno-fetal transmission risk*		
Differential cost^a^	-78	
Event differential per 1000 women screened^a^	0.16	5.07
Cost per additional outcome avoided	474,296	15,331
*75% reduction of materno-fetal transmission risk*		
Differential cost^a^	-77	
Event differential per 1000 women screened^a^	0.84	5.74
Cost per additional outcome avoided	92,027	13,494
***Antenatal Screening Participation Rate***		
*50% participation rate*		
Differential cost^a^	-49	
Event differential per 1000 women screened ^a^	0.21	3.27
Cost per additional outcome avoided	232,636	14,826
*100% participation rate*		
Differential cost^a^	-97	
Event differential per 1000 women screened^a^	0.42	6.55
Cost per additional outcome avoided	232,642	14,826

^a:^ Cost and event differential are calculated following the same formula: results with the neonatal screening minus results with the prenatal screening

**Table 5:** Sensitivity analyses: Long term assessment (Toxoscreen project).

Sensitivity analyses	Strictly *Toxoplasma-* Related Events	Global Events
***Epidemiological context***		
*Northern European country or United States (prevalence*: *10%*, *incidence*: *0*.*05%)*		
Differential cost	-113	
Event differential per 1000 women screened^a^	0.01	3.46
Cost per additional outcome avoided	9,107,766	32,724
*Southern European countries (prevalence*: *50%*, *incidence*: *1%)*		
Differential cost	-55	
Event differential per 1000 women screened^a^	0.14	3.67
Cost per additional outcome avoided	406,308	15,276
***Spiramycin Efficacy***		
*25% reduction of the materno-fetal transmission*		
Differential cost	-76	
Event differential per 1000 women screened^a^	0.02	3.49
Cost per additional outcome avoided	3,311,013	21,625
*75% reduction of the materno-fetal transmission*		
Differential cost	-75	
Event differential per 1000 women screened^a^	0.1	3.51
Cost per additional outcome avoided	754,215	21,302
***Antenatal screening participation rate***		
*50% participation rate*		
Differential cost	-47	
Event differential per 1000 women screened^a^	0.03	2.19
Cost per additional outcome avoided	1,795,488	21,472
*100% participation rate*		
Differential cost	-94	
Event differential per 1000 women screened^a^	0.05	4.38
Cost per additional outcome avoided	1,788,429	21,473

^a^: Cost and event differential are calculated following the same formula: results with the neonatal screening minus results with the prenatal screening

In order to evaluate what might have been the results in other epidemiological contexts, we estimated the results using the prevalence and the incidence of seroconversion in non-immune women in northern European countries or in the United States. In the short-term assessment (one year), the cost per additional GE avoided was € 23,168. The higher difference in costs compared to the base-case may be explained by the higher number of women to screen (90%) due to low prevalence (i.e. 10%). Indeed, the cost of the prenatal screening was € 893 in this scenario (vs € 850 in the base-case analysis) whereas the cost of neonatal screening was only slightly modified (€ 777 vs € 773 in the base-case analysis) as well as the events’ differential. In this context, the 15-year cost per additional GE avoided increased to €32,724 (including long-term neurological and ophthalmologic sequelae).

Conversely, in a country of higher prevalence, such as Middle to Southern European countries, where prevalence can be as high as 50% and incidence of infection in non-immune women may be as high as 1%, the 1-year ICER and the 15-year ICER were reduced to €9,830 and €15,276 per avoided GE, respectively. In this context, the higher incidence of maternal infection and the higher cost of care for infected children were balanced by the much lower number of women at risk to screen (50%).

Tables 4 and 5 provide also the results of the sensitivity analysis for variations in spiramycin efficacy in preventing materno-fetal transmission. The efficacy of spiramycin needed to reach 75% to allow the 1-year ICER to reach less than €100,000 per avoided STRE, but including global events the ICER varied little and stayed below €15,331 throughout the range of efficacy. When considering the 15-year assessment, the cost per avoided GE was €21,625 even with a 25% reduction of the materno-fetal transmission rate by spiramycin.

Very few data are available on the prenatal screening participation rate. We relied on the data published in Rhône-Alpes for the base-case analysis (80% participation rate [46]). This rate, however, may be lower or higher in other regions. Thus, we considered a 50% to 100% range of variation for this parameter. The lower the participation rate, the lower is the likely number of avoided events by prenatal screening vs neonatal strategy. We found, however, that the impact of variations in screening participation rates was trivial: the 15-year cost per avoided GE stayed around € 21,472 whatever the participation rate.

Of note, the only variable that induced major change in the 1-yr and 15-year ICERs is the specificity of neonatal screening test. Indeed, when accounting for the lower value retrieved in the literature, i.e. 92%, the 1-yr and 15-yr cost per avoided GE were reduced to €4,458 and €6,519, respectively (not shown in Table 4). Conversely, the sensitivity and specificity of the prenatal tests had almost no impact on the results, nor did a 20% efficacy of the prenatal pyrimethamine/sulfadiazine treatment.

## Discussion

Our objective was to assess the effectiveness and costs of neonatal screening in France compared to the current prenatal program. We built a decision-analysis model that 1) reflected French guidelines and practices over 20 years [13], 2) included the most recent knowledge on mother-child toxoplasmosis transmission and its long-term consequences in treated children, and 3) allowed for various epidemiological contexts in terms of incidence, prevalence, treatment efficacy, and adherence to the compared strategies.

Compared to the prenatal strategy, neonatal screening was found to be less effective and also less expensive. Prenatal screening was cost-effective when considering all adverse events globally. For the 15-year endpoint, the cost per avoided GE remained below €33,000; for the 1-year endpoint, the cost per avoided GE was below €23,500. French Gross Domestic Product per capita is €38,026, which some consider relevant as the threshold below which a strategy may be considered efficient [53]. ICERs remained below this threshold for GE in all sensitivity analyses.

French health-care professionals and the general public are accustomed to the mandatory screening for toxoplasmosis that has been in place for almost 30 years in France. But falling incidence and the pressure of other health priorities could lead French policy makers to look for other options. Among the few countries that have any screening program at all, neonatal screening is the second most common practice, the first being prenatal screening.

Because neonatal programs have been implemented in other settings, we chose to compare the French practice of prenatal screening to neonatal screening. To our knowledge, no study has compared the cost-effectiveness of these two strategies. Two published studies have compared prenatal screening with no systematic screening and found that screening was cost-saving in the low-prevalence setting of the United States [54] and the moderate-prevalence setting of Austria [55]. Our results are in agreement with these studies; indeed, screening for infected newborns at birth excludes the possibility of intervention during pregnancy and consequently does not prevent events such as fetal infection, fetal injuries, fetal loss, pregnancy termination, and neonatal death. However it must be recognized that prenatal screening and treatment of infected women and children reduces the risk of transmission and sequelae [5,56] but does not eliminate all risks as shown by Wallon et al in 2013 [5].

One limit of this study is related to the fact that we did not account for the psychological consequences involved with prenatal or neonatal screening. Identifying a pregnant woman as non-immune allows her to take the right measures to avoid infection, but this daily need to pay strict attention may be stressful, as may be monthly retesting. Identification of an acute infection can generate feelings of guilt and stress in this context. But it offers the opportunity to treat the infection, adapt the follow-up, and potentially reduce the risk of disability in infected children and related distress in parents. Accounting for those psychological consequences should have thus reinforced our results. Given the possible adverse events (visual impairment, mental retardation but also pregnancy termination or fetal loss due to amniocentesis), a cost-utility analysis should be of major interest. However, at the present we have no data on the preferences of parents as well as of infected children and teenagers related to each possible outcome in this context. Further studies are thus required to allow assigning each adverse event a reliable value in terms of quality adjusted life years.

Another limitation is that, in the absence of randomized intervention trials, we could only rely on indirect estimates for the efficacy of prenatal treatment, but uncertainty regarding their exact benefits was accounted for by the sensitivity analysis. It showed that spiramycin had to reduce the transmission probability of 75% to reduce the 1- year ICER below €100,000, when considering the STRE. Conversely, variations in prenatal pyrimethamine/sulfadiazine (PS) treatment marginally modified the base-case results. In addition, variations in prenatal treatment efficacy (spiramycin and PS) had little impact on results when other events were also considered (GE). We did not include the possibility that treating postnatally infected children with the combination of pyrimethamine and sulphonamides might also reduce sequelae. Such additional impact would only have further increased the difference between the two strategies’ efficacy.

Finally, our results are based on risk estimates for mother-child transmission and for sequelae that were obtained in a setting where Type II strains predominate. In a context of higher virulence, the French protocol of prenatal screening should be even more cost-effective, because preventing transmission or sequelae would have greater preventive effects. In addition, a higher parasitic pressure is associated with higher prevalence among pregnant women and lower proportion of women to be retested during pregnancy.

Further benefits of the French practice of prenatal screening for toxoplasmosis are very likely much more extensive than is recognized. Monthly visits and testing raise the overall standard of care, providing opportunities to increase the education of mothers and to recognize early signs of potential problems of pregnancy. As one example, it has been hypothesized that the higher incidence of listeriosis reported in southwest France might be the indirect consequence of higher prevalence of toxoplasmosis in the area, requiring fewer women to be tested and thus repeatedly reminded to pay attention to their food-, hand- and kitchen-hygiene [57]. However, it must be acknowledged that detecting pregnancy abnormalities earlier through a closer follow-up does not eliminate pregnancy problems, particularly the ones unrelated to toxoplasmosis (e.g. fetal losses occurring in the 1^st^ trimester); it can only help in initiating the diagnostic process earlier, in the case of ultrasound abnormalities and give time to a shared decision between parents and health care professionals on the pregnancy outcome.

Another indirect benefit is the unique contribution that existing prenatal screening programs have made to clinical research. A large proportion of the publications on the management of toxoplasmosis were based on data provided from screening programs. Moreover, much remains to be understood regarding risk factors and the best diagnostic strategies and treatment. These improvements would benefit not only those who are screened, but also mothers and babies who are diagnosed worldwide as the result of individual screening performed at the initiative of patients or physicians.

Finally another putative benefit is the prevention of possible neuropsychiatric disorders that could be due to both postnatal and congenital infection. It has indeed been recently hypothesized that postnatal and congenital infection might be associated with late onset neurological or psychiatric consequences, which would also be worth preventing [58].

Screening success relies heavily on the compliance of practitioners and patients. In France, consciousness of toxoplasmosis is deeply integrated in health care professional practice and in the popular medical culture. Indeed, education begins early in the basic training of health care professionals and benefits from a network of reference centers that are available for questions from laboratory professionals, gynecologists, or midwives, particularly on serology interpretation as well as on treatment follow-up. In addition, pregnant women can be regularly reminded of food-related risks by health care professionals, as well as by other women among their family and friends but also by mass media [59]. Implementing such a screening program in a country without such a deeply ingrained consciousness of the disease would require efforts to train health care professionals and to provide information directly to women and the public generally.

Reducing costs and constraints of screening is another significant line of research. The impact of using new diagnostic tests to be integrated into existing strategy at the laboratories or as Point-of-Care tests [60], including individual rapid fingerprick [61] or saliva tests [62], is currently being investigated. Being able to rely on a prenatal screening program is key to investigating, in real life conditions without selection bias, such new diagnostic strategies, or for performing additional clinical research. It offers unique opportunities for testing new ways of using existing drugs and for testing the impact of new treatment strategies while accounting for major confounders, such as the gestational age at maternal infection. It also provides the chance to collect updated unbiased estimates of prevalence and incidence data, and to understand better how pregnant women get infected.

In addition, globalization and new trade agreements between Europe and South America may expose the French population to a wider genetic diversity of the parasite through, for example, imported meat. Climate change may also impact the parasite distribution, combined with change in eating habits with more organic food direct from farmers. These changes may expose women more frequently to *T gondii* and potentially to more virulent parasites, inducing more severe symptoms in children and reinforcing the cost-effectiveness of the prenatal screening strategy.

## Conclusion

To conclude, our study demonstrated the efficiency of prenatal screening compared to neonatal screening. It was based on an elaborate decision tree that could be adapted to other settings as a reliable tool for ranking the main options for reducing the burden of congenital toxoplasmosis. The French experience demonstrates that prenatal screening is feasible and cost-effective compared to neonatal screening and worth being pursued in France.

## Supporting information

S1 Appendix Tree structure. Fig A: General scheme of the decision tree; Fig B1: Subunit of the decision tree describing the neonatal screening when no abnormality is discovered during the usual ultrasound evaluation of pregnant women; Fig B2: Subunit of the decision tree describing the neonatal screening when an ultrasound abnormality is discovered during the usual ultrasound follow-up of pregnant women and the fetus is infected; Fig B3: Subunit of the decision tree describing the neonatal screening when an ultrasound abnormality is discovered during the usual ultrasound follow-up of pregnant women and the fetus is not infected; Fig C1: Subunit of the decision tree describing the first events accounted for in the context of prenatal screening; Fig C2: Subunit of the decision tree describing prenatal screening when maternal seroconversion is not recognized; Fig C3: Subunit of the decision tree describing prenatal screening when maternal seroconversion is recognized by prenatal screening and no ultrasound abnormality is discovered; Fig D1: Subunit of the decision tree describing the events that may occur in children with recognized symptomatic congenital toxoplasmosis (SCT); Fig D2: Subunit of the decision tree describing the events that may occur in children with recognized asymptomatic congenital toxoplasmosis (ACT); Fig D3: Subunit of the decision tree describing the events that may occur in children with unrecognized symptomatic congenital toxoplasmosis; Fig D4: Subunit of the decision tree describing the events that may occur in children with unrecognized asymptomatic congenital toxoplasmosis; With Congenital Toxoplasmosis: CT; Symptomatic Congenital Toxoplasmosis:SCT; Asymptomatic Congenital Toxoplasmosis: ACT; True Positives: TP; False positives: FP; True Negatives: TN, False Negatives: FN.
(PDF)

S2 Appendix French protocol. (PDF)

S3 Appendix Literature review. (PDF)

S4 Appendix Lyon cohort. Organization for case identification and data collection; Flow-chart of the final mother and child populations in the study (Source: Wallon M, Peyron F, Cornu C, Vinault S, Abrahamowicz M, Bonithon-Kopp C, et al. Congenital toxoplasma infection: monthly prenatal screening decreases transmission rate and improves clinical outcome at age 3 years. Clin Infect Dis 2013;56(9):1223‑31).
(PDF)

S5 Appendix Cost hypotheses. (PDF)
