# Supplementary material for: The cost-effectiveness of neonatal versus prenatal screening for congenital toxoplasmosis
Source: PLoS One. 2019 Sep 18;14(9):e0221709. doi: 10.1371/journal.pone.0221709 (PMC6750576; doi:10.1371/journal.pone.0221709)

S1-Tree Structure

The structure of the decision tree can be split into different subunits. The first part describes the course of the pregnancy and the status of the child at birth. The second part, describing the post-natal outcomes for congenitally infected children, is itself divided into subunits corresponding to the various contingencies. The first part of the tree, taking into account the usual medical follow-up of women during pregnancy, is divided into three subunits, each corresponding to one trimester of pregnancy. The starting point of each subunit corresponds to a decision node from which two branches leave: 1) branch 1: birth screening; 2) branch 2: prenatal screening. We took into account the fact that some women in the regime of neonatal screening may benefit from an elective prenatal screening. Similarly, adherence to antenatal screening is considered imperfect. In this context, however, a serological examination should be carried out at birth and the children should be screened in the same way as if a neonatal screening had been planned. S1 Figure 1A summarizes the general pattern of the tree and the link between the different subunits mentioned below.

S1 Figure A: General scheme of the decision tree (Toxoscreen project)

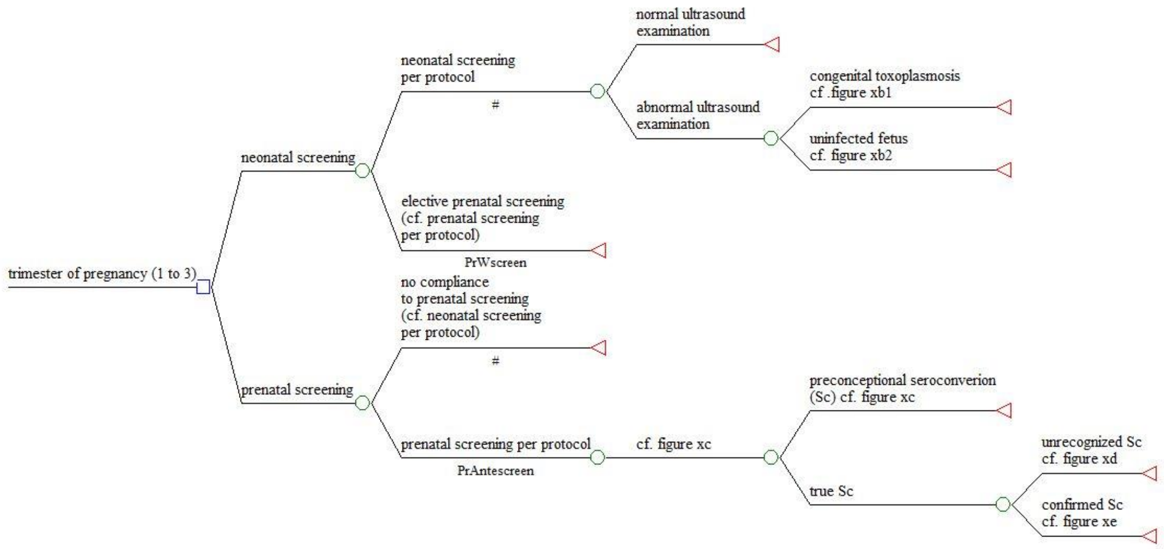

a. Screening at birth

Neonatal screening is the first subunit. We considered that, in the French context, pregnancy monitoring includes three ultrasound examinations, which could be the occasion for the discovery of a fetal abnormality. In the absence of an abnormality discovered during the ultrasound monitoring, the decision tree is represented in S1 Figure 2A. In the context of screening at birth, in the absence of ultrasound lesions and serologically suggestive CT, a routine check-up will be carried out at birth, allowing only the most severe forms of Symptomatic Congenital Toxoplasmosis (SCT) to be recognized. Conversely, when the neonatal screening is positive, a specific toxoplasmosis examination is performed at birth allowing a better identification of symptomatic forms of CT than the routine check-up.

The terms appearing above each branch describe the contingencies described. The terms below each branch correspond to the names of the variables defining the probabilities assigned. Some abbreviations should be defined. Thus, SCT corresponds to a symptomatic form of CT either clinically (hepato-splenomegaly, microcephaly for example) or paraclinically (calcification, chorioretinitis, etc). ACT (asymptomatic congenital toxoplasmosis) corresponds to an asymptomatic form of CT manifested only by abnormal laboratory results. The abbreviation TP describes the true positives, FN, false negatives, FP, false positives and TN true negatives.

S1 Figure B1: Subunit of the decision tree describing the neonatal screening when no abnormality is discovered during the usual ultrasound evaluation of pregnant women (Toxoscreen project)

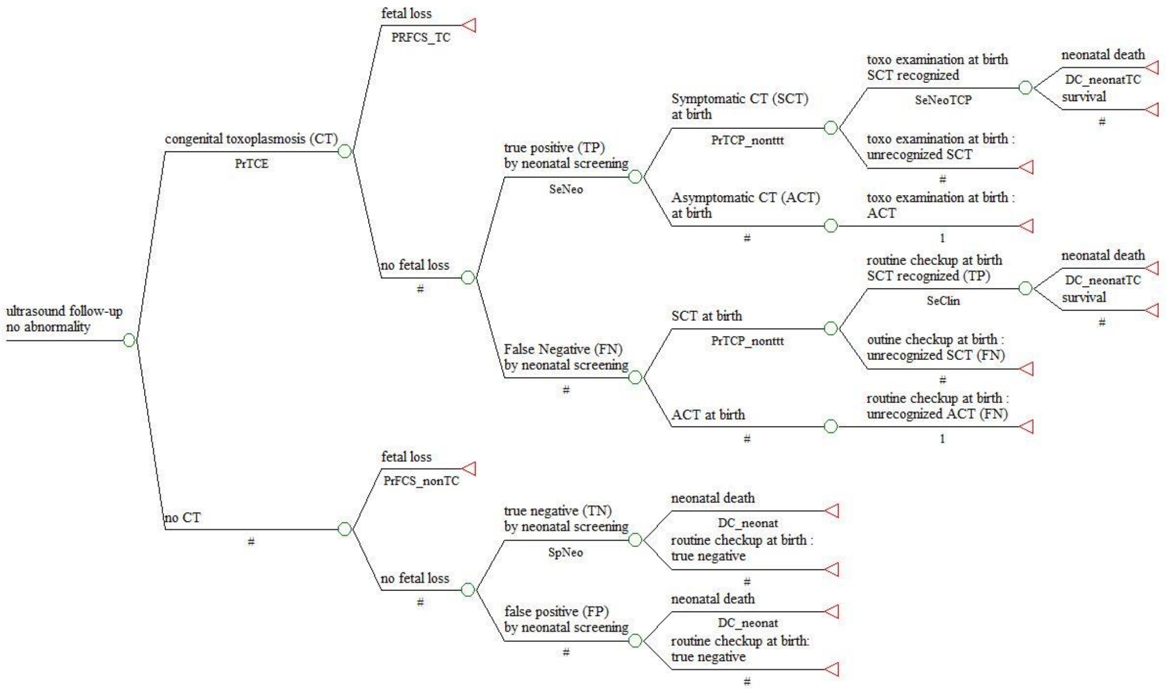

In Denmark, the discovery of a fetal anomaly only very rarely generated a diagnosis of congenital toxoplasmosis (personal communication by E Petersen); conversely, we considered that the discovery of such an abnormality in France may lead to the diagnosis of CT because of the greater awareness of toxoplasmosis among health professionals and the general public. Indeed, the long history of the policy of prevention against toxoplasmosis (30 years), the inclusion of this infection as a specific item in the initial training program of medical doctors and midwives, as well as the widespread information about this risk on websites and fora dedicated to pregnant women lead to a shared culture of awareness about congenital toxoplasmosis. Thus, if ultrasound surveillance reveals an anomaly, two possibilities are taken into account, in line with the observations made in pilot surveys among the 30 French university hospital centres conducted in the early 2000s (Binquet *et al*. Presse Med 2004). The surveys used dedicated postal questionnaires on knowledge, attitude, and practices about congenital toxoplasmosis and were followed up by a similar study among 1556 Burgundy general practitioners (participation rate=25%), private pediatricians (n=79; participation rate=14%) and obstetricians (n=94; participation rate=34%) (Binquet C. PhD thesis, University of Burgundy, 2003). The two possibilities were:

- termination
- amniocentesis allowing an antenatal diagnosis in an etiological research procedure.

In the latter case, the fetus can either be infected or not. The consequences of these two possibilities are described in S1 Figures 2B and 2C.

S1 Figure B2: Subunit of the decision tree describing the neonatal screening when an ultrasound abnormality is discovered during the usual ultrasound follow-up of pregnant women and the fetus is infected (Toxoscreen project)

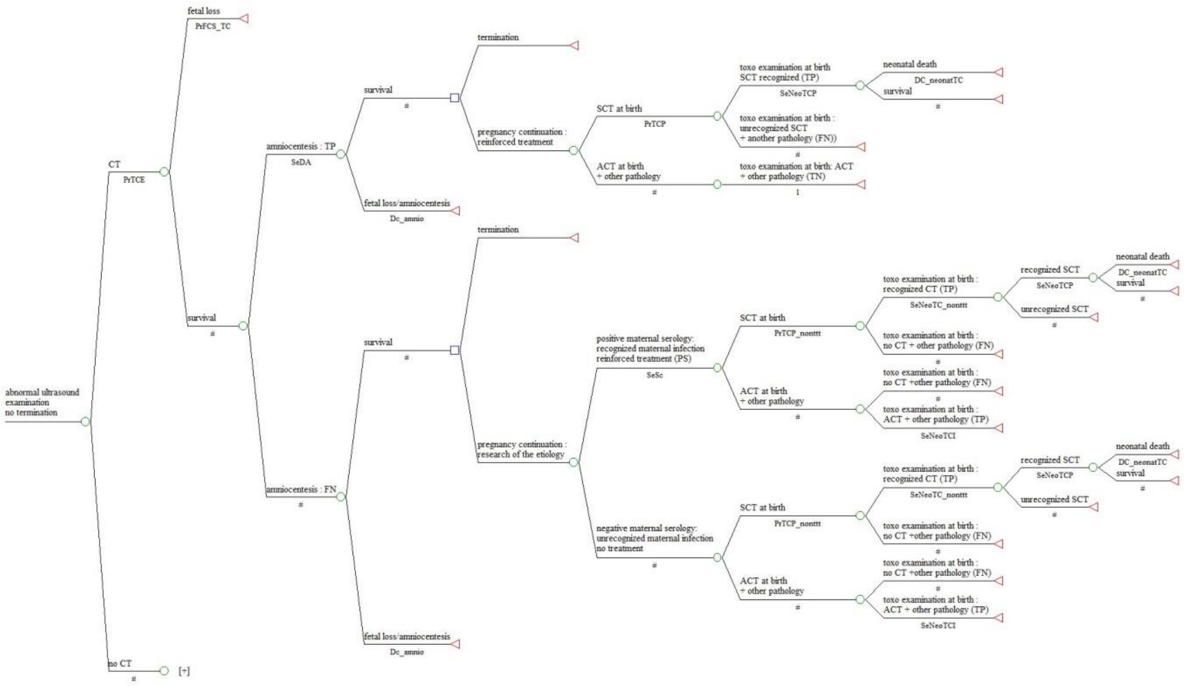

The first assumption is that in the case of a positive prenatal diagnosis (amniocentesis), the child is considered to be infected, even if this is not the case. While the mother was not treated by spiramycin, the child is prenatally and postnatally treated as if he had CT with pyrimethamine and sulfadiazine; then the evolution of the serology during the first year of life allows the refutation of this diagnosis. In this context of abnormal ultrasound examination, we include the possibility of termination, whether the prenatal diagnosis was positive or not, because the severity of the ultrasonographic lesions alone could warrant termination for some people according to the results of the pilot surveys mentioned above. In addition, in the event of an abnormal ultrasound examination, the etiological assessment includes not only amniocentesis, but also the realization of maternal serology (CT serology) that may or may not confirm maternal infection, which further guides the therapeutic decision of treating the mother with pyrimethamine and sulfadiazine (reinforced treatment). Chronologically the realization of maternal serology is performed before amniocentesis. We considered, however, that amniocentesis would be carried out systematically in the context of an etiological assessment. This examination being a primary element of the diagnosis, the inversion of the chronological order allowed us to simplify the structure of the tree slightly without changing the final result.

Then, if CT is suspected, a check-up should be routinely performed at birth or in the neonatal period because of the absence of early diagnosis and to differentiate, rightly or wrongly, between SCT and ACT. In the context of screening at birth, in the absence of ultrasound lesions and serologically suggestive CT, a routine check-up will

be carried out at birth, allowing only the most severe forms of SCT to be recognized. Moreover, we considered that the neonatal assessment (toxoplasmosis neonatal examination) and the routine check-up at birth had 100% specificity. Moreover, neonatal death can only be observed in the case of SCT if the symptoms are severe. In this case those symptoms are specific enough to make CT recognized. Whatever the screening regime the probability of the fetus being infected is directly related to seroprevalence, incidence, proportions of seroconversion during the pregnancy trimesters, and the risk of mother-to-child transmission during that period.

S1 Figure B3: Subunit of the decision tree describing the neonatal screening when an ultrasound abnormality is discovered during the usual ultrasound follow-up of pregnant women and the fetus is not infected (Toxoscreen project)

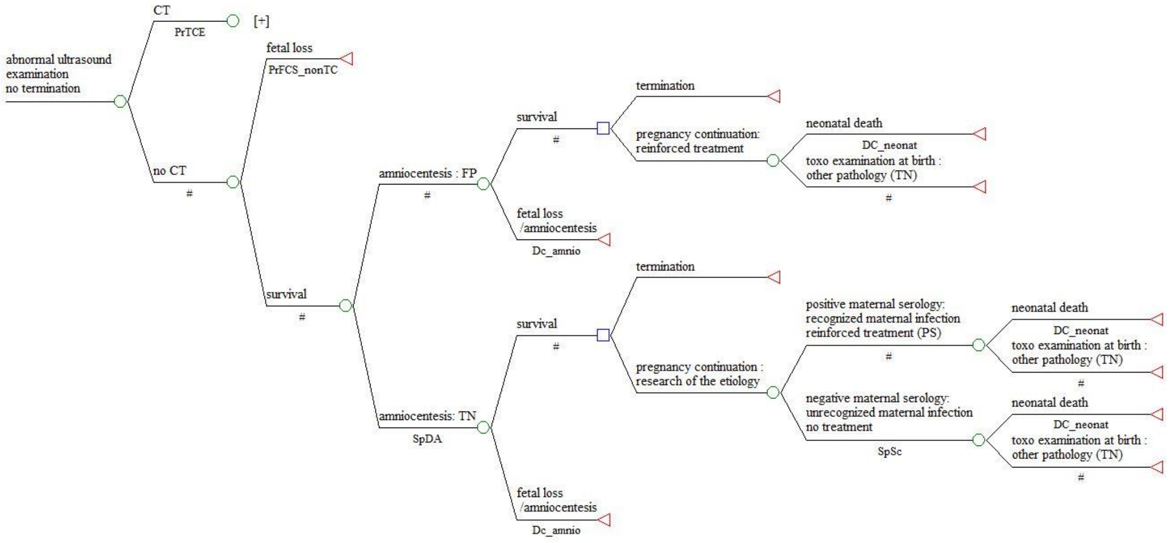

b. Prenatal screening

S1 Figure 3A describes the main events occurring under a program of prenatal screening. In the case of preconceptional seroconversion that is wrongly recognized as a true seroconversion, we thought it prudent, considering the answers to the pilot surveys mentioned above (Binquet *et al*. *Presse* Med 2004; Binquet PhD thesis 2003) to account for the possibility of a termination although it is not recommended. If the pregnancy is pursued, the situation is the same as if the seroconversion is confirmed, but the child will not be infected.

S1 Figure C1: Subunit of the decision tree describing the first events accounted for in the context of prenatal screening (Toxoscreen project)

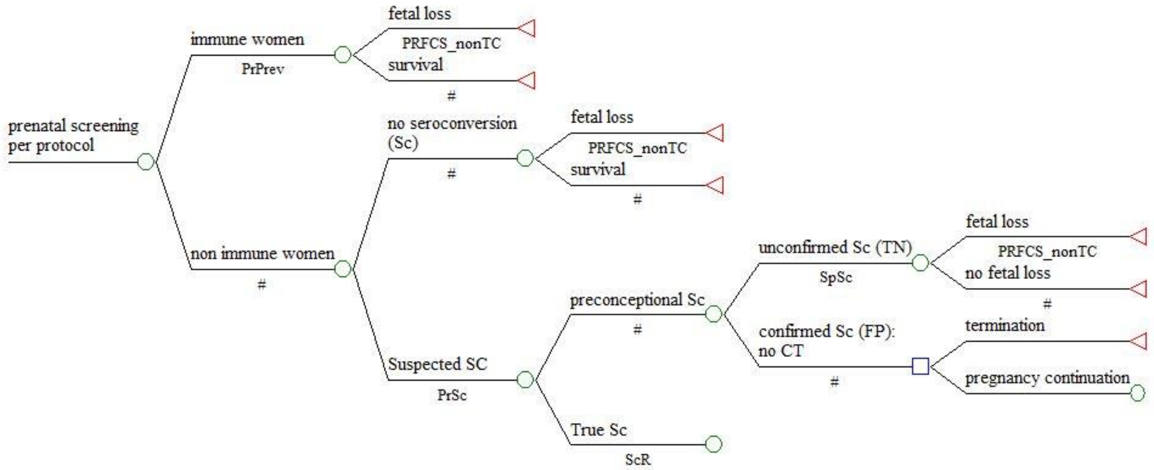

In the case of a true seroconversion, it can either be recognized or not recognized (S1 Figure 3B). It is important to take this latter situation into account since this phenomenon is particularly frequent for maternal infection during the 8th and 9th months of pregnancy. In this case, the assessment that should be made at delivery and/or in the neonatal period makes it possible to identify some infections. When the maternal seroconversion is not recognized, the fetus may or may not be infected, and ultrasound monitoring may or may not show a fetal abnormality. In the case of abnormal ultrasound examination, management was considered to be similar to that described in the context of neonatal screening with an abnormal ultrasound examination during the usual routine ultrasound monitoring of pregnant women (three recommended ultrasound examinations).

S1 Figure C2: Subunit of the decision tree describing prenatal screening when maternal seroconversion is not recognized (Toxoscreen project)

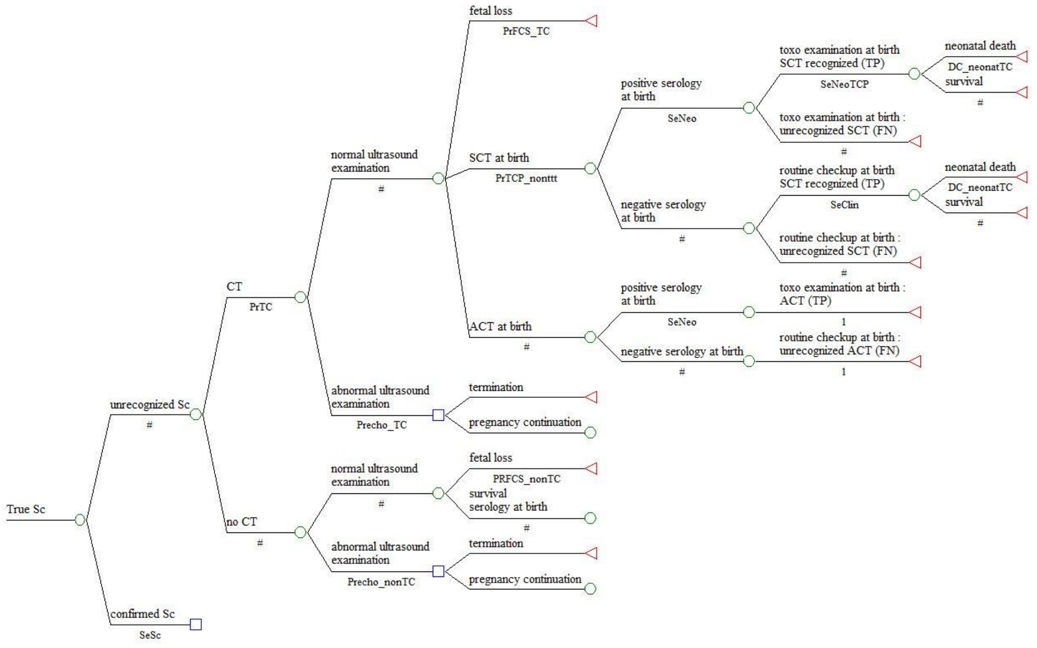

When an actual seroconversion is confirmed, some practitioners may propose a pregnancy termination as shown by our pilot study (Binquet *et al*., *Presse* Med 2004). Thus we accounted for this choice. In the absence of termination, several cases are possible depending on whether the child is infected and/or whether ultrasound monitoring is able to detect an abnormality. In the case of abnormal ultrasound examination, termination may be proposed. If this option is not favored, our pilot surveys led us to consider several therapeutic options:

- Initiation of treatment with spiramycin as soon as seroconversion is discovered; then amniocentesis is performed as soon as possible, and enhanced treatment (PS) is given in the case of positive prenatal diagnosis and/or further abnormal ultrasound examination;
- Initiation of treatment with a pyrimethamine-sulfonamide (PS) combination (reinforced treatment), without amniocentesis. This regimen cannot be prescribed before the 16th week of pregnancy, so treatment with spiramycin precedes the reinforced treatment in case of 1st trimester maternal infection;
- Initiation of PS treatment as soon as possible and amniocentesis. If the latter is positive and/or a lesion is observed on ultrasound examination, the reinforced treatment is maintained until delivery; otherwise treatment with spiramycin is resumed and maintained until delivery.

If ultrasound monitoring does not reveal any particular abnormality, a final option is considered. It consists in ultrasound follow-up along with the prescription of spiramycin until delivery. The figure below (S1 Figure 3C) shows these different options in the case of a truly infected child, in the absence of fetal abnormalities on ultrasound.

One can notice that, in the context of a recognized seroconversion, a complete neonatal assessment (toxo examination at birth) is considered at delivery or in the neonatal period. It makes it possible most of the time to differentiate ACT and SCT; even some false positives and false negatives may be observed.

S1 Figure C3: Subunit of the decision tree describing prenatal screening when maternal seroconversion is recognized by prenatal screening and no ultrasound abnormality is discovered (Toxoscreen project)

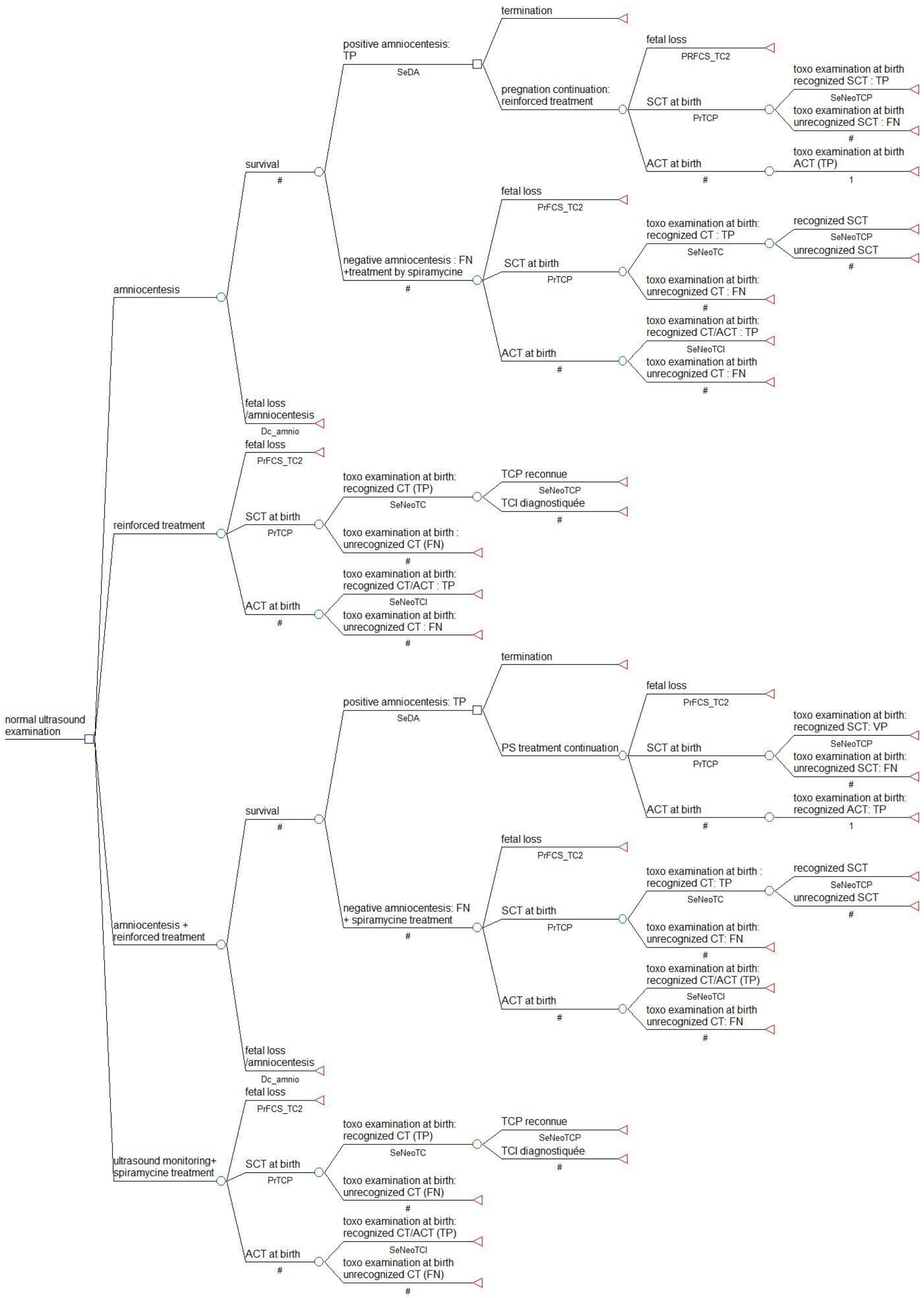

c- Follow-up of children until 15 years of age

c-1. Recognized CT

If the management of the pregnant woman and of the child at birth leads to CT recognition, two possible outcomes are considered. If the child is suffering from SCT, his follow-up could be illustrated in the decision tree represented below (S1 Fig 4). In the case of a confirmed SCT with a retinochoroiditis discovered at birth, whatever the activity of the lesion, the child is treated during the first year of life with pyrimethamine and sulfonamides. As the probability of sequelae is not higher in the case of an active lesion (see below), we have chosen to simplify the decision tree by not differentiating active and inactive ocular lesions at birth.

S1 Figure D1: Subunit of the decision tree describing the events that may occur in children with recognized symptomatic congenital toxoplasmosis (SCT - Toxoscreen project)

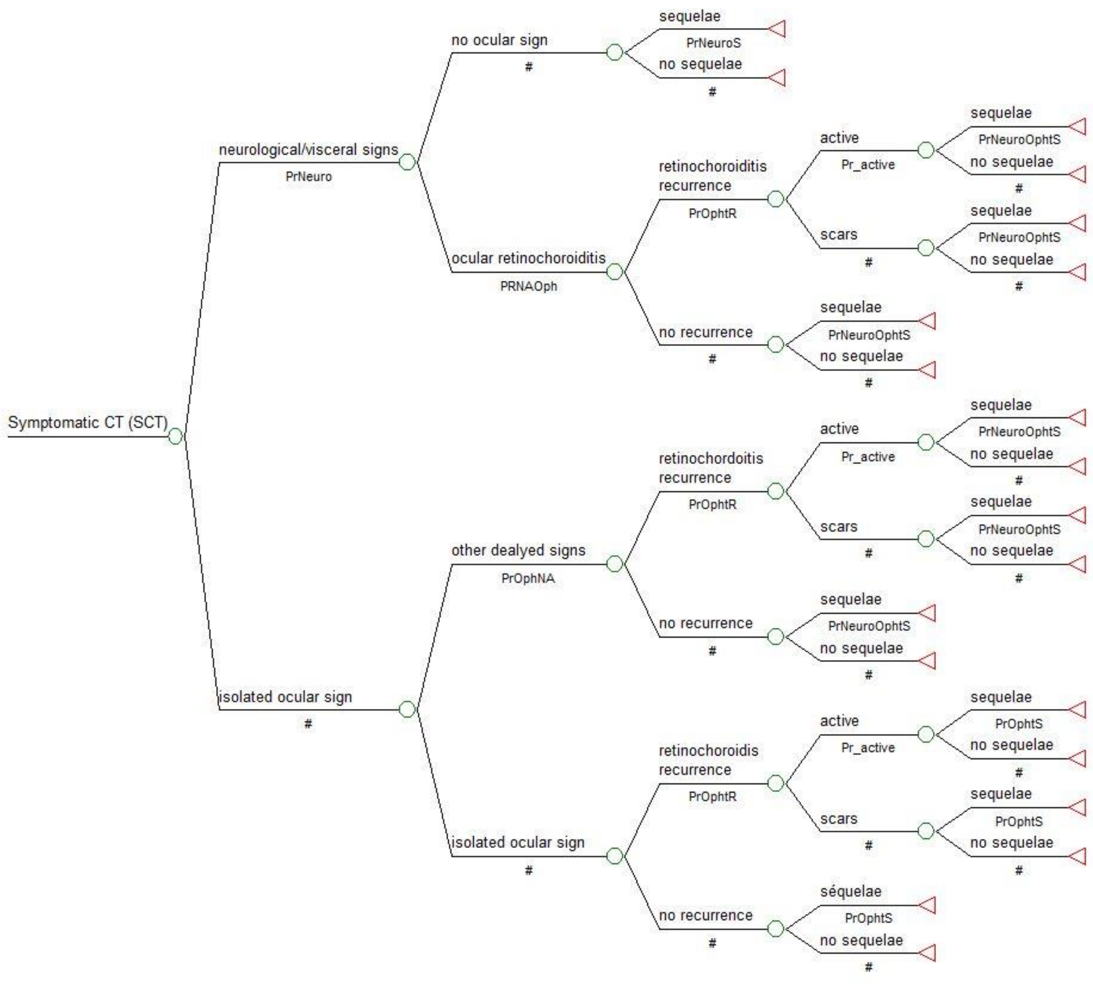

If the child presents with ACT at birth, the corresponding decision tree is represented by Figure 4B. The first year of care is identical in both cases.

S1 Figure D2: Subunit of the decision tree describing the events that may occur in children with recognized asymptomatic congenital toxoplasmosis (ACT - Toxoscreen project)

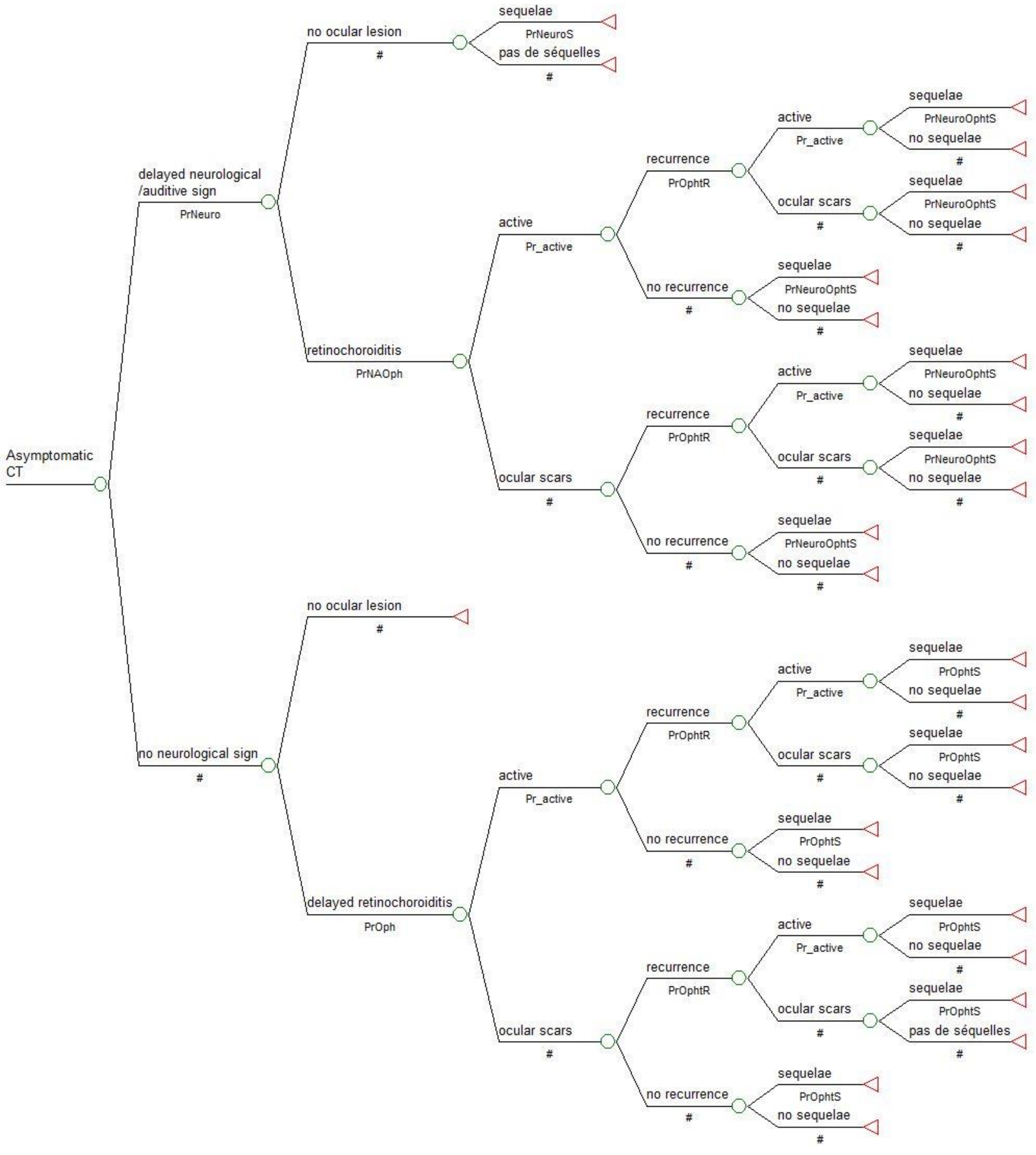

c-2. Suspected but unconfirmed CT at birth

In the case of suspected CT:

- if the child is actually infected, we consider that follow-up is similar to that of children for whom the infection is confirmed, apart from a lag of a few months after which the diagnosis is confirmed since the serologies are systematically repeated to confirm or invalidate the diagnosis of infection;

- If the child is actually not infected, the serological follow-up during the first year of life refutes the diagnosis of CT.

c-2. Unidentified CT

If CT is not suspected, the previous trees are slightly modified to account for the lack of recognition of CT signs (S1 Figures 4C and 4D). We based the follow-up of children on the protocol established by the Lyon and Marseille centers (S2).

S1 Figure D3: Subunit of the decision tree describing the events that may occur in children with unrecognized symptomatic congenital toxoplasmosis (Toxoscreen project)

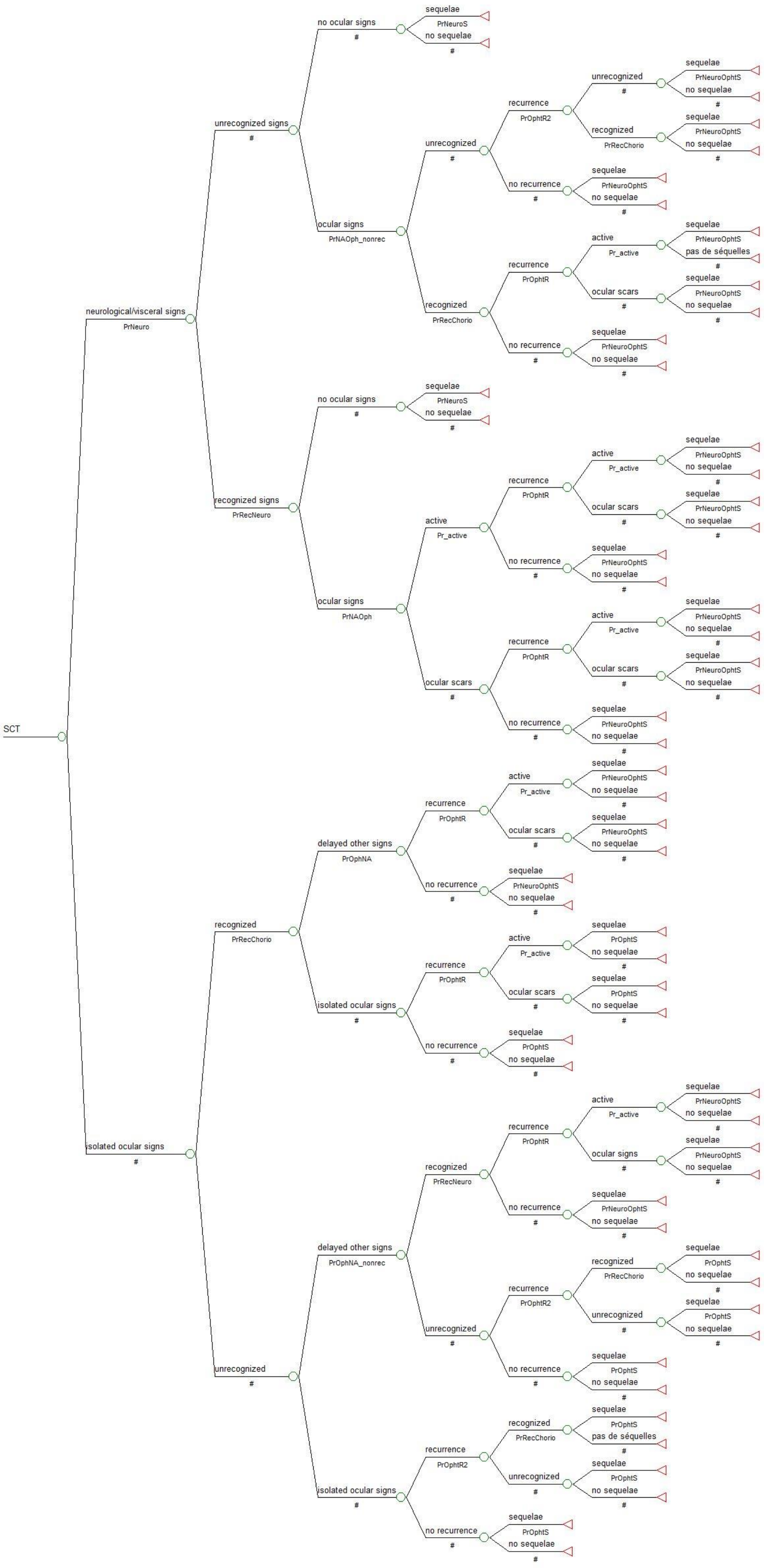

S1 Figure D4: Subunit of the decision tree describing the events that may occur in children with unrecognized asymptomatic congenital toxoplasmosis (Toxoscreen project)

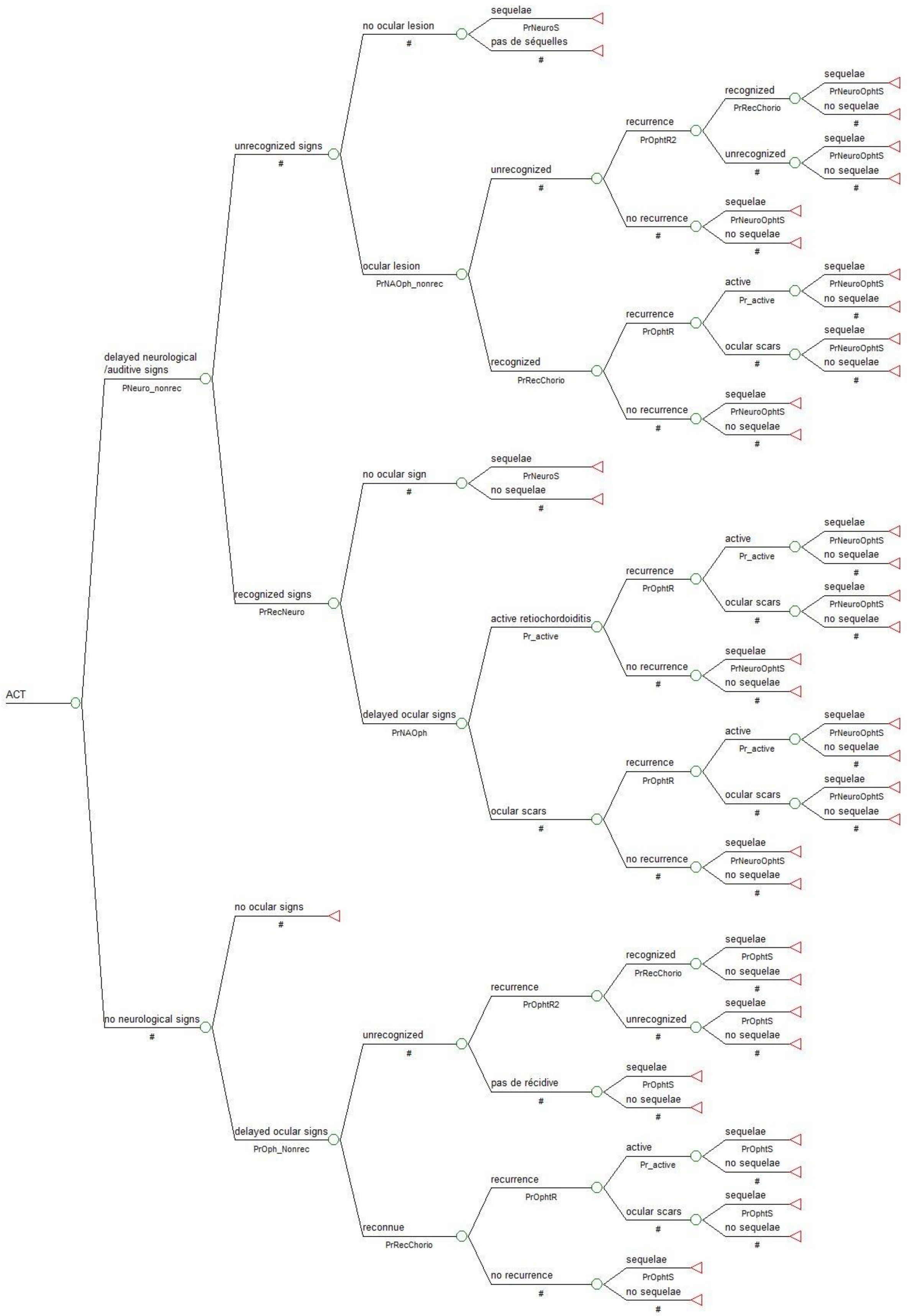

Supplement: S1 Appendix — Fig A: General scheme of the decision tree; Fig B1: Subunit of the decision tree describing the neonatal screening when no abnormality is discovered during the usual ultrasound evaluation of pregnant women; Fig B2: Subunit of the decision tree describing the neonatal screening when an ultrasound abnormality is discovered during the usual ultrasound follow-up of pregnant women and the fetus is infected; Fig B3: Subunit of the decision tree describing the neonatal screening when an ultrasound abnormality is discovered during the usual ultrasound follow-up of pregnant women and the fetus is not infected; Fig C1: Subunit of the decision tree describing the first events accounted for in the context of prenatal screening; Fig C2: Subunit of the decision tree describing prenatal screening when maternal seroconversion is not recognized; Fig C3: Subunit of the decision tree describing prenatal screening when maternal seroconversion is recognized by prenatal screening and no ultrasound abnormality is discovered; Fig D1: Subunit of the decision tree describing the events that may occur in children with recognized symptomatic congenital toxoplasmosis (SCT); Fig D2: Subunit of the decision tree describing the events that may occur in children with recognized asymptomatic congenital toxoplasmosis (ACT); Fig D3: Subunit of the decision tree describing the events that may occur in children with unrecognized symptomatic congenital toxoplasmosis; Fig D4: Subunit of the decision tree describing the events that may occur in children with unrecognized asymptomatic congenital toxoplasmosis; With Congenital Toxoplasmosis: CT; Symptomatic Congenital Toxoplasmosis:SCT; Asymptomatic Congenital Toxoplasmosis: ACT; True Positives: TP; False positives: FP; True Negatives: TN, False Negatives: FN. (PDF) [file pone.0221709.s001.pdf]
