# Supplementary material for: The cost-effectiveness of neonatal versus prenatal screening for congenital toxoplasmosis
Source: PLoS One. 2019 Sep 18;14(9):e0221709. doi: 10.1371/journal.pone.0221709 (PMC6750576; doi:10.1371/journal.pone.0221709)
Supplement: S2 Appendix — (PDF) [file pone.0221709.s002.pdf]

S2-French protocol

At each subsequent monthly assessment, *Toxoplasma* infection is defined as the appearance of specific IgG antibodies. Specific IgM and IgG antibodies are detected using a selection of commercialized tests. In case of infection, treatment with spiramycin (3g daily) is initiated immediately, monitoring is increased (from trimestral to monthly ultrasound examination), and fetal infection is identified using polymerase chain reaction (PCR) of amniotic fluid. When PCR is positive, the mother is treated with pyrimethamine (50 mg daily) and sulfadiazine (1.5 g twice daily) until delivery. Interruption of pregnancy is discussed when severe abnormality is discovered on ultrasound examination. The same drug combination is also prescribed presumptively for third-trimester infections, as an alternative to fetal blood or amniotic fluid sampling. In absence of compliance with prenatal screening, the newborn can benefit at least from neonatal screening.

Assessment at birth includes cerebral ultrasonography, ocular examination, and serological testing of neonatal blood for IgM, IgA, and IgG. Treatment is started at birth or as soon as infection is confirmed and comprises two regimens: 1) pyrimethamine (P) and sulfadiazine (S) up to the age of > 2 months or body weight > 5 kg, given continuously (P: 1 mg/kg/day; S: 50 mg/12 h); and then 2) Fansidar® (P: 1.25 mg/kg/10 days; and sulfadoxine: 25 mg/kg/10 days) for a further 10–12 months. All children of mothers with primary infection in pregnancy undergo a pediatric check-up and an assessment of neurological development, as well as serological testing for IgG and IgM, every 3 months for one year. Neurological, ophthalmological, and serological examinations are repeated at least once a year with no age limit. When an active retinal lesion is detected after termination of treatment, Fansidar® is resumed for a further 3 months.

Serologies are performed in medical laboratories that may or may not be connected to hospitals and confirmed when necessary by reference university hospital laboratories. When the first test is positive for IgG, quantification of IgM antibodies is performed as well as avidity testing in order to determine whether infection occurred before or after conception. Doubt may arise regarding recent infection, in which case additional tests are required (including avidity). Insufficient attention to parental anxiety and inadequate education by doctors may lead parents to opt for abortion (which is legal in France before the 12th week of gestation without any medical justification) for fear of potential future disability.
