# Supplementary material for: The cost-effectiveness of neonatal versus prenatal screening for congenital toxoplasmosis
Source: PLoS One. 2019 Sep 18;14(9):e0221709. doi: 10.1371/journal.pone.0221709 (PMC6750576; doi:10.1371/journal.pone.0221709)
Supplement: S3 Appendix — (PDF) [file pone.0221709.s003.pdf]

S3-Literature Review

A review of the literature was carried out using Medline, Embase, Biological Abstract and Pascal. The following strategy was used for the probabilities related to prenatal and neonatal screening as well as to prenatal toxoplasmosis-related events:

1 - the combination of the key words "toxoplasmosis" and "congenital" was used in key words and in free text;

2 - the articles on toxoplasmosis in animals and those in a language other than French, English, German, Dutch, Spanish or Italian have been excluded from this selection;

3 - the abstracts of all selected articles were systematically read;

4 - articles that reported original data were read in full and the probabilities corresponding to the outcomes of interest were extracted.

The central estimate of each probability was determined according to the most recent and valid original report. The range of variation was defined according to the minimum and the maximum found in the literature, whatever the quality of the studies.

Specific research was also performed for unrelated toxoplasmosis events, such as overall or unrelated fetal loss rate, overall neonatal deaths, fetal abnormalities as well as performance of ultrasound examination for detecting such abnormalities using the same databases but specific keywords; original, English-language papers with the best level of evidence were selected for the estimation of those probabilities.

The materno-fetal transmission rates, the distributions of symptomatic and asymptomatic cases at birth, as well as the post-natal toxoplasmosis-related events were estimated from the TOXO-LY cohort and were mostly published elsewhere (Wallon M et al. Pediatrics 2014;133(3):e601-608; Wallon M et al. Clin Infect Dis 2013;56(9):1223-31)

When data were not available, we relied on the opinion of three experts i.e. :E Petersen, R Piarroux, and M Wallon for defining the central estimate of unknown probabilities as well as their possible range of variation; E Petersen gave estimates for the probabilities concerning neonatal screening; R Piarroux and M Wallon built a consensus for prenatal screening probabilities, prenatal and post-natal treatment efficacy, as well as the performance of toxo examination at birth during a specific meeting aimed at validating the tree structure.
