# Supplementary material for: The cost-effectiveness of neonatal versus prenatal screening for congenital toxoplasmosis
Source: PLoS One. 2019 Sep 18;14(9):e0221709. doi: 10.1371/journal.pone.0221709 (PMC6750576; doi:10.1371/journal.pone.0221709)
Supplement: S4 Appendix — Organization for case identification and data collection; Flow-chart of the final mother and child populations in the study (Source: Wallon M, Peyron F, Cornu C, Vinault S, Abrahamowicz M, Bonithon-Kopp C, et al. Congenital toxoplasma infection: monthly prenatal screening decreases transmission rate and improves clinical outcome at age 3 years. Clin Infect Dis 2013;56(9):1223‑31). (PDF) [file pone.0221709.s004.pdf]

S4-Lyon Cohort

Organization for case identification and data collection

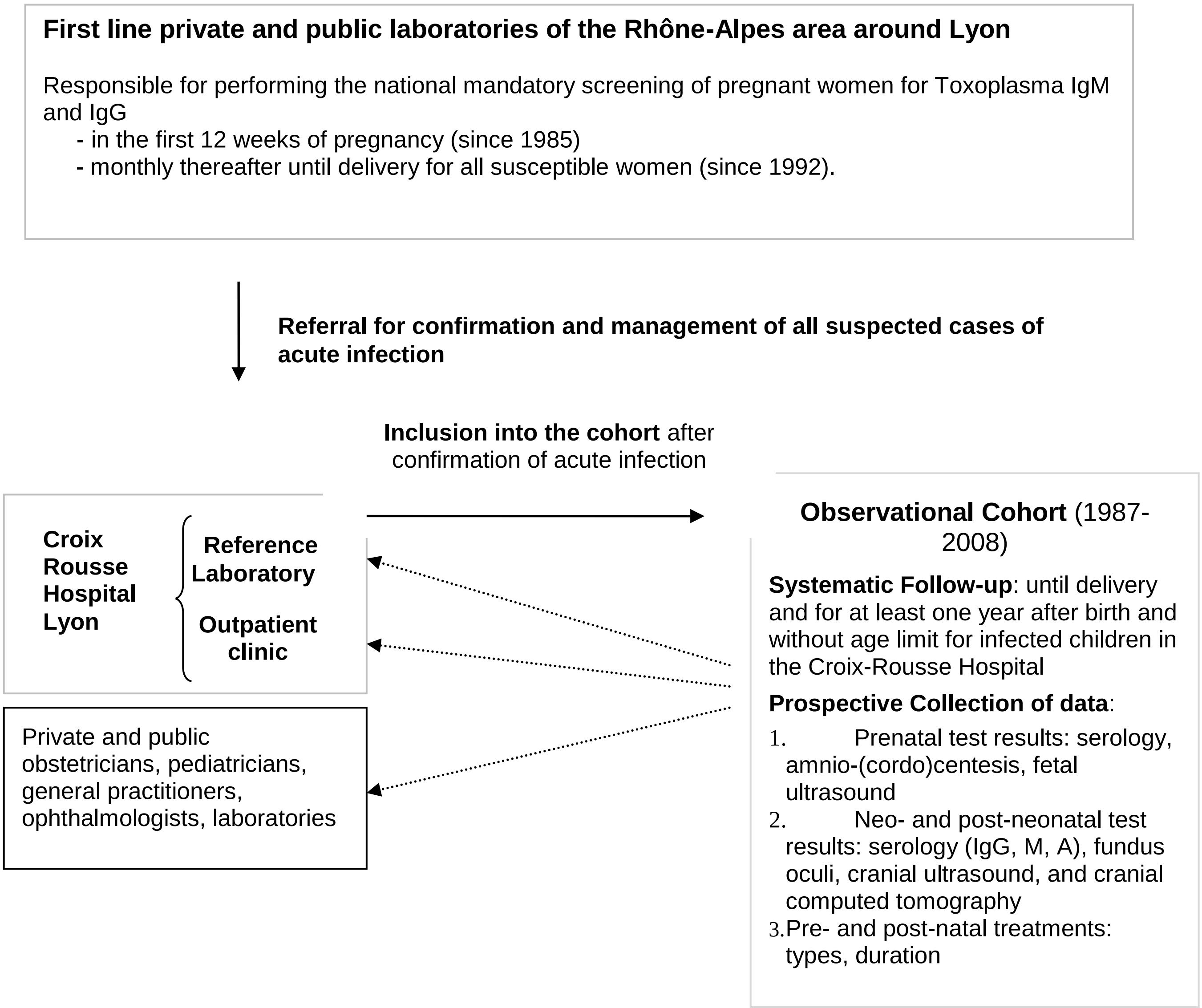

Source : Wallon M, Peyron F, Cornu C, Vinault S, Abrahamowicz M, Bonithon-Kopp C, et al. Congenital toxoplasma infection: monthly prenatal screening decreases transmission rate and improves clinical outcome at age 3 years. Clin Infect Dis 2013;56(9):1223-31.

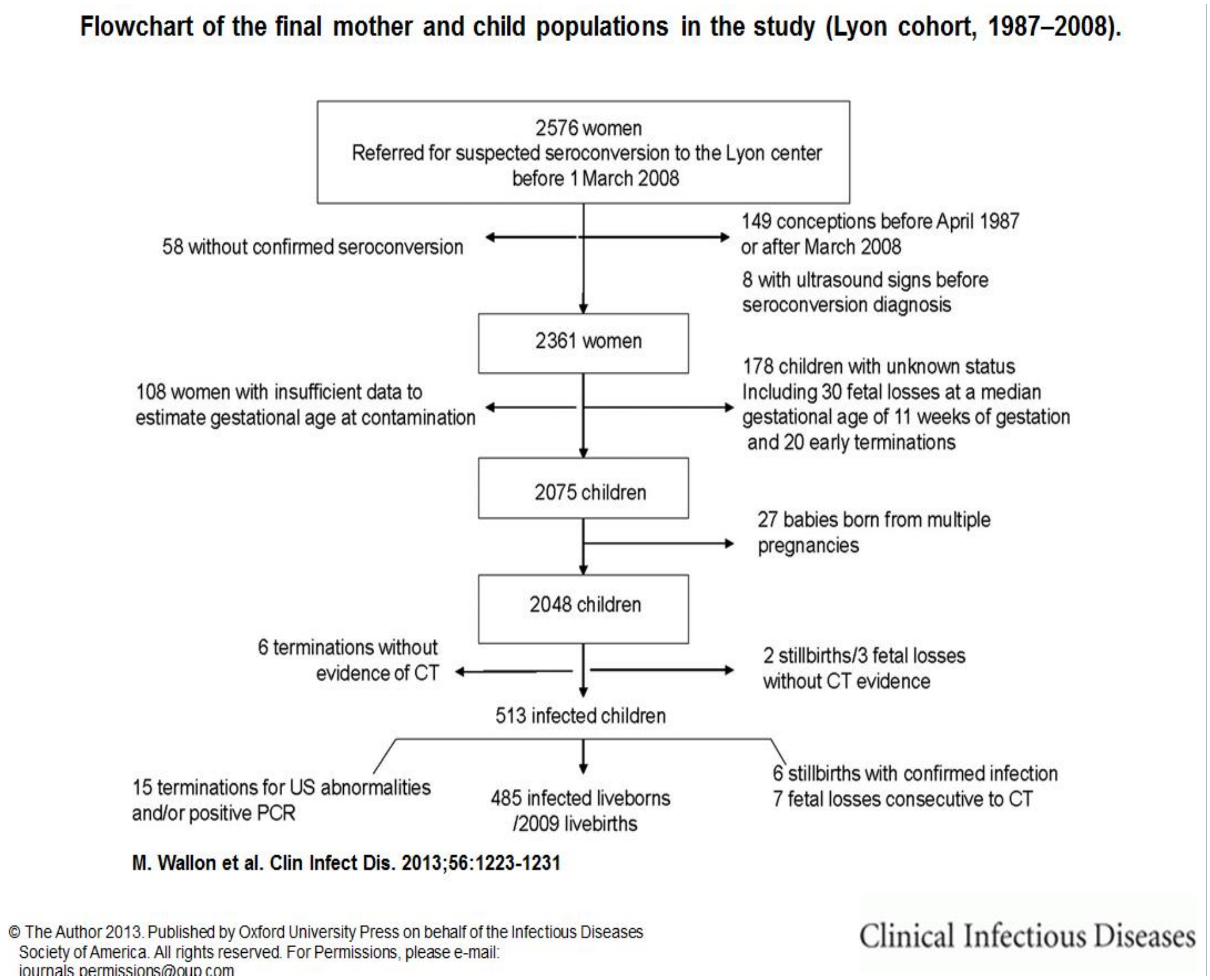

Source : Wallon M, Peyron F, Cornu C, Vinault S, Abrahamowicz M, Kopp CB, et al. Congenital toxoplasma infection: monthly prenatal screening decreases transmission rate and improves clinical outcome at age 3 years. Clin Infect Dis 2013;56(9):1223‑31.
