# Supplementary material for: The cost-effectiveness of neonatal versus prenatal screening for congenital toxoplasmosis
Source: PLoS One. 2019 Sep 18;14(9):e0221709. doi: 10.1371/journal.pone.0221709 (PMC6750576; doi:10.1371/journal.pone.0221709)
Supplement: S5 Appendix — (PDF) [file pone.0221709.s005.pdf]

S5-Cost Hypotheses

Preamble: Prenatal care and neonatal care are particularly well covered in France by the National Health Insurance (NHI). Specifically, the mandatory prenatal examinations (seven medical visits and the monthly laboratory examinations including screening for toxoplasmosis) are fully covered by the NHI using a third-party payment system whatever the private or public status of the health care professional (obstetrician, general practitioner, or midwife) performing such examinations. Ultrasound (US) examinations performed from the 6th month of pregnancy on are also fully covered by NHI. Previous recommended ultrasound examinations (2 US exams have to be performed before the end of the 5th month) are reimbursed by the NHI, but 30% of their tariff is reimbursed by complementary health insurance. If any pregnancy-related pathology is detected, clinical visits as well as complementary examinations are fully covered.

All neonatal check-ups are fully covered by the NHI until 12th day of life and until 28 days for children with a confirmed infection. After this period and in children with a suspected infection, the rate of examinations coverage is 60% and when the infection is confirmed, 65% of treatment costs are reimbursed by the NHI; for 95% of the French population, the amount to be paid after reimbursement is usually covered by a supplementary insurance. In case of symptomatic toxoplasmosis, full coverage by the NHI of all the medical costs required for the child's care may be claimed.

Cost hypotheses: Direct costs were calculated on the basis of: 1) the French protocol detailed in Part 2 and 2) pilot surveys carried out with the parasitology departments of the French university hospitals and private practitioners published elsewhere (1).
Different assumptions were required to estimate the cost associated with each of the branches of the decision tree. The presentation of these hypotheses is detailed for each of the branches:
- prenatal period,
- childbirth and neonatal period,
- follow-up of children to 15 years of age.

1. Prenatal part of the decision tree

In the absence of intervening events, childbirth is assumed to occur after 38 weeks and 4 days of pregnancy (total gestation period of 270 days).

1.1. Dates of infection

As explained in Part 1, the consequences of maternal infections are modeled in three different subtrees according to their trimester of occurrence. The assumption was made that infection occurs at the midpoint of each of the considered trimesters. Thus, first trimester infections are hypothesized to occur during the 7th week of pregnancy (9th week of amenorrhoea), second-trimester infection during the 20th week of pregnancy (22nd week of amenorrhoea) and the third Infection during the 33rd week of pregnancy (35th week of amenorrhea).

1.2. Fetal loss

a. In the case of congenital toxoplasmosis

According to the Toxo-Ly cohort, for maternal infection during the first trimester, fetal loss occurs on average 11 weeks (interquartile range [IQR]: 8–13 weeks) after infection. Fetal loss would therefore occur on average during the 18th week of pregnancy (IQR: 15.5–20.5 weeks). For infection during the 2nd trimester (infection during the 20th week of pregnancy), the fetal loss occurs on average 10 weeks after the date of infection (IQ: 9–10 weeks). Fetal loss would therefore occur on average during the 30th week of pregnancy (IQ: 28–30.5 weeks).

b. In the absence of congenital toxoplasmosis

Fetal losses are assumed to occur in the middle of each trimester i.e. during the 7th week when the first trimester is concerned, during the 20th week for the second trimester and during the 30th week for the third trimester.

1.3. Serological monitoring

All laboratory tests are reimbursed according to a score relying on a key letter (B) and a coefficient. Corresponding fees are calculated by multiplying this latter coefficient by the price given to the letter by the National Health Insurance system. Concerning Toxoplasmosis, a serological examination that includes the identification and titration of at least two different immunoglobulin isotypes (including IgG) by at least two different techniques corresponds to a B40 score and to a cost of €10.80. This score is applied for the initial testing as well as for subsequent follow-up. In case of seroconversion (positive serology following a previous negative serology) or of a significant increase in the level of antibody *Toxoplasma*, a B60 score (€16.20) is applied for seroconversions during the 2nd and 3rd trimester. It corresponds to an examination and a control on a new sample in the event of a limit rate, a suspicion of recent infection or of a seroconversion, by at least two immunoglobulin (IgG) isotypes by at least two different techniques and this rating includes simultaneous titration of the previous serum. In particular cases of doubt about the initial immune status of women, which is particularly frequent for maternal infection during the 1st trimester, a second B60 may also be accounted for.

Given the monthly screening, we calculated that non-immune pregnant women without infection or fetal loss would have 7 serological tests (all scored B40). In addition to those serologies, given the difficulty that may be encounter to ensure that women are truly seronegative or positive at the beginning of the pregnancy, we added an additional serology (B40) at the end of the 2nd month for 25% of cases. In case of a fetal loss unrelated to congenital toxoplasmosis occurring during the 1st trimester we considered that no serological test would be performed. Conversely for fetal losses occurring during the 2nd trimester, 3 tests would be carried out, and for 3rd trimester fetal losses, 5 tests have to be performed. The following table (S5 table 1) presents the number of serological tests when a seroconversion is detected.

S5 Table 1. Number of serological tests administered in non-immune women when a maternal seroconversion is suspected because of maternal serology modification (not fetal abnormalities)

Number of serological tests	without transmission		with transmission	
	no fetal loss	fetal loss	no fetal loss	fetal loss
Serconversion during				
1st trimester	1[a]	0	1[a]	1[a]
2nd trimester	3+1[a]	3	3+1[a]	3+1[a]
3rd trimester	5+1[a]	4	5+1[a]	-

a. a B60 score is considered for each serology which aims at confirming a suspected seroconversion.

1.4. Ultrasound monitoring

a. In the absence of fetal loss

The usual monitoring of pregnancy includes the completion of three ultrasound examinations. The first examination is ideally performed at 10 weeks of pregnancy (WP) and aims to establish the age of pregnancy and identify a multiple pregnancy. The corresponding score according to the classification Commune des Actes Medicaux (CCAM) of the National Insurance System is JNQM001 with a cost of €36.35. The second is realized around 19 WP. It is a morphological ultrasound examination that aims to detect fetal abnormalities and is rated JQQM018 (€100.2). The third ultrasound exam is performed during the 8th month (30 WP) to determine the delivery conditions and is rated JQQM016 (€100.2). In the event of detection of seroconversion, a monthly ultrasound is usually performed. As soon as seroconversion is recognized, the monthly ultrasound monitoring would be a morphological ultrasound exam. Referring to the date estimates of infection reported above, we considered that infection of the 1st trimester occurred during the 7th WP. The seroconversion would then be discovered during the control performed during the 3rd month, when an ultrasound is already planned. A monthly ultrasound monitoring is then carried out over the remaining period, i.e. over 6 months. Thus, 7 ultrasound exams could be performed in this case (1 JNQM001 and 3 JQQM018 and 3 JQQM016). We hypothesized that a seroconversion of the 2nd trimester occurs during the 20th WP. Seroconversion is diagnosed in this case at best during the 6th-month assessment, at about 25 WP. The mother will have had two ultrasound examinations before the diagnosis of toxoplasmic seroconversion (1 JNQM001 and 1 JQQM018) and monthly ultrasound monitoring in the remaining months of pregnancy. In this case, 6 ultrasound exams are accounted for (1 JNQM001 and 2 JQQM018 and 3 JQQM016). Finally, for a seroconversion of the third trimester occurring during the 33rd WP, three ultrasounds are performed in any case (1 JNQM001 and 1 JQQM018 and 1 JQQM016). The discovery of seroconversion will be made at the earliest at the end of the 8th month or at the beginning of the 9th month. An ultrasound will then be carried out, bringing the number of ultrasounds to 4 (1 JNQM001 and 1 JQQM018 and 2 JQQM016). In summary, the number of ultrasounds would be 7 for an infection of the 1st trimester, 6 for an infection of the 2nd trimester and 4 for an infection of the 3rd trimester.

b. Accounting for fetal losses / Interruption of pregnancy

In the event of fetal loss and termination of pregnancy, the number of additional ultrasounds should be reduced compared to ultrasound monitoring in cases of recognized or suspected CT. The number of ultrasounds would be reduced to two (1 JNQM001, 1 JQQM018) for an infection during the first trimester (range of variation 2–3), and to three (1 JNQM001, 2 JQQM018) for an infection occurring during the 2nd trimester of pregnancy (range of variation: 2–4). If a termination is performed following the discovery of ultrasound lesions before confirmation of the fetal infection, it is hypothesized to be carried out during the 16th week of pregnancy for an infection of the first trimester. According to the same hypothesis, the termination in case of seroconversion during 2nd trimester would be carried out during the 27th week of pregnancy. Thus the number of ultrasounds is identical in this case, as described above in the case of fetal loss.

S5 Table 2. Number of ultrasound examinations in case of maternal infection

	Identified seroconversion		Unrecognized seroconversion[a]	
	No fetal loss	Fetal loss	No fetal loss	Fetal loss
Time of Seroconversion				
1st trimester	7	2	3	1
2nd trimester	6	3	3	3
3rd trimester	4	-	3	-

a. similar situation in case of congenital infection and neonatal screening

In the absence of seroconversion and / or fetal infection, 12.5% of pregnancies end in fetal loss during the first trimester. We assume that no ultrasound will be performed in this case. We also considered that 2.5% of pregnancies end in the second trimester. These pregnancies benefit at most from two ultrasounds (1 JNQM001, 1 JQQM018), except in case of recognition of a seroconversion; in this case, there will be 1 ultrasound scored JNQM001 and 1 or 2 ultrasound examinations scored JQQM018. Marginally (0.43%), fetal losses can also occur in the 3rd trimester. These pregnancies have benefited at most from 3 ultrasound examinations (standard follow-up).

1.5. *Amniocentesis*

Amniocentesis is performed at the earliest during the 16th week of pregnancy; the results of the PCR in amniotic fluid are usually obtained within 15 days following the amniocentesis. It must be performed at least 4 weeks after the date of maternal infection. Given our assumptions, in the absence of fetal infection, if a fetal loss is observed during the first trimester, no amniocentesis can be performed.

We accounted for the fact that fetal loss due to amniocentesis may occur in the days following amniocentesis. Furthermore, if a termination was decided given a positive PCR, we hypothesized that this termination was performed within the two weeks following the PCR results.

S5 Table 3. Summary of hypothesized dates of infection, seroconversion discovery, amniocentesis, and fetal losses (weeks of pregnancy)

	1st Trimester	2nd Trimester	3rd Trimester
Maternal infection	6.5	19.5	32.5
Seroconversion			
Identification	9.5	24.5	35
Confirmation	12	—	—
Amniocentesis	16	25	35.5
PCR results	17	26	36.5
Fetal loss	17.5	29.5	—

1.6. Therapeutic management and treatment monitoring

a. <u>In the case of fetal infection with or without fetal loss</u>

Treatment with spiramycin is initiated as soon as seroconversion is discovered. Spiramycin is usually prescribed at 3 million units (MU) three times a day. This treatment is maintained until the change to a reinforced treatment or until delivery when simple medical supervision (including monthly ultrasound monitoring) is preferred or when the amniocentesis is negative and no ultrasound abnormality is discovered. This reinforced treatment can be prescribed using two forms:

- pyrimethamine (Malocide ®) tablet at 50 mg daily, combined with sulfadiazine (Adiazine ®) at two 500 mg tablets three times a day;
- pyrimethamine combined with sulfadoxine at 1 tablet per 20 kg every 10 days.

The most common approach in France is to recommend the prescription of pyrimethamine and sulfadiazine; thus this hypothesis was retained for estimating the cost of CT prevention strategies. In all cases, a concomitant prescription of folinic acid (Lederfoline ®) is recommended (2 tablets at 25 mg every 7 days). This reinforced treatment may be prescribed in case of positive PCR, but may be also prescribed as a 1st line treatment as soon as it is possible, that is after the 15th WP. The treatment durations are summarized in S5 Tables 4a and 4b.

S5 Table 4a. Summary of spiramycin treatment duration (weeks) according to the trimester during which the maternal infection occurred (treatment duration in the case of fetal loss is presented in parentheses)

	Negative PCR leading to medical supervision	Positive PCR leading to reinforced treatment	1st line reinforced treatment*
1st trimester	30.5 (7.5)	6.5 (6.5)	5 (5)
2nd trimester	16.5 (5)	1.5 (1.5)	0 (0)
3rd trimester	6	1.5	0

* This first line reinforced treatment corresponds to the immediate prescription of pyrimethamine/sulfadiazine +folinic acid as soon as a seroconversion is confirmed

S5 Table 4b. Summary of pyrimethamine/sulfadiazine treatment duration (weeks) according to the trimester during which maternal infection occurred (treatment duration in the case of fetal loss is presented in parentheses)

	Negative PCR medical supervision	Positive PCR reinforced treatment	1st line reinforced treatment

1st trimester	0	23 (1.5)	25 (2)
1st trimester	0	14.5 (3.5)	16 (5)
3rd trimester	0	4	5.5

In the case of ultrasound abnormalities, if a termination is not elected, a reinforced treatment is initiated as soon as these lesions are discovered. This treatment is maintained until delivery. The initiation of a pyrimethamine / sulfadiazine treatment requires the completion of a blood count before 1st treatment administration and every 15 days thereafter. If adiazine / malocide is prescribed, proteinuria should be measured weekly. The number of proteinuria tests performed is therefore identical to the number of weeks of treatment. The usual monitoring of pregnancy includes a blood count, performed during the 6th month of pregnancy and a monthly monitoring of proteinuria. The table below (S5 Table 5) summarizes the total number of blood counts and proteinuria measurements that were included in the analysis.

S5 Table 5. Summary of blood counts and proteinuria measurements (in parentheses) that should be performed during pregnancy when no fetal loss occurs

	Negative PCR medical supervision	Positive PCR reinforced treatment	1st line reinforced treatment
1st trimester	1/0 (7/2)	12/1 (23/2)	7/2 (25/2)
2nd trimester	1/0 (7/5)	8/3 (15/4)	5/3 (16/5)
3rd trimester	1 (7)	3 (7)	4 (6)

b. <u>In the absence of fetal infection with or without fetal loss</u>

Considering the 1st trimester subtree, when a maternal infection is suspected, but without transmission to the fetus or in the absence of maternal infection, if a fetal loss occurs, it is assumed to occur during the 1st trimester. No amniocentesis will be performed. At most, an assessment including an ultrasound and a proteinuria measurement will be realized. In the absence of fetal loss, management of the seroconversion either rightly or wrongly identified will be similar to the one described in part a. If the maternal seroconversion is not recognized or does not occur, the usual monitoring of pregnancy applies, including a blood count in the 6th month and monthly proteinuria testing.

For a maternal infection suspected during the 2nd trimester, without transmission to the fetus or in the absence of maternal infection, if a fetal loss occurs, it is assumed to occur in the middle of the 2nd trimester. In the absence of maternal infection, between two and three proteinuria measurements may be performed and one or two ultrasound examinations. In case of maternal infection without transmission to the fetus, there will not be time to identify this infection. No additional examination or treatment is therefore considered, compared to the usual monitoring. In the absence of fetal loss, in the absence of maternal infection, or in cases of unrecognized maternal infection, the routine monitoring of pregnancy is used in the analysis. If a maternal infection is identified (rightly or wrongly), without transmission to the fetus, the management of a seroconversion occurring in the 2nd trimester is applied.

For maternal infection suspected in the 3rd trimester, without transmission to the fetus (which in any case will not have time to be identified) or in the absence of maternal infection, if a fetal loss occurs, it occurs before 30th

week of pregnancy. In the absence of infection, 5 proteinuria controls will be performed at most, as well as a blood count and two ultrasound examinations. In the absence of fetal loss, infection or in cases of unrecognized maternal infection, routine monitoring of pregnancy is applied. In case of recognized maternal infection (rightly or wrongly), without transmission to the fetus, the management of seroconversion in the 3rd trimester applies.

1.7. Neonatal screening

Routine pregnancy monitoring for all patients consists of three ultrasound examinations, one blood count, and monthly proteinuria testing. In the case of 1st trimester infection, if the fetal infection induces an abnormality identifiable on ultrasound, it will be identified during the fetal ultrasound carried out in the 5th month (during the 20th WP). The etiological assessment including serology and amniocentesis will be carried out within the next 15 days and the definitive results known at the earliest between the 23rd week and the 25th week (2 serological tests at 3 week interval, plus time to get PCR results). On average, it is similar to a 2nd trimester infection recognized in the prenatal screening framework, with initiation of reinforced treatment in the case of positive PCR. In the case of negative PCR, if the maternal infection has been identified, the scenario is similar to a recognized 2nd trimester seroconversion. In the absence of recognition of maternal infection, the situation is equivalent to the search for any other pathology. In case of fetal loss due to toxoplasmosis, which occurs during the 18th week of pregnancy, only one ultrasound will have been performed, as well as two proteinuria controls.

In case of a 2nd trimester infection, if the fetal impairment induces an ultrasound abnormality, it will be recognized during the 3rd trimester assessment carried out during the 30th week of pregnancy. Making the same assumptions as above, the results of the investigation will be recognized during the 34th week of pregnancy (serology results at the earliest during the 33rd WP). If PCR is positive, 6 weeks of enhanced treatment preceded by a week of spiramycin are included in the analysis, as well as 3 ultrasound examinations, 4 blood counts, and 11 proteinuria controls. In the case of a negative PCR, if the maternal infection has been identified, we considered that the same scenario applies. If the maternal infection is not recognized, another pathology is sought. In the case of fetal loss, it occurs during the 29th week and therefore only two ultrasounds and 4 proteinuria controls are counted. Finally, in the case of 3rd trimester infection, we considered that the probability of an abnormal ultrasound examination related to CT is nil. In that case, the usual monitoring is included.

2. At birth

2.1 Neonatal screening

The cost of neonatal screening is based on the assumption that a “classical” serology is performed (B60) in newborns; no data on neonatal screening tests are sufficiently detailed in the literature to support another hypothesis.

2.2 Toxoplasmosis examination at birth or during the neonatal period

The neonatal assessment includes the following examination:

- Skull radiography,
- Cranial ultrasonography,
- Ocular examination (Fundus),
- Placenta mouse inoculation + PCR and cell culture,
- serology for the newborn (IgG, IgM and IgA in the peripheral blood),
- serology for the mother (IgG, IgM).

As the clinical examination of the children is carried out in any context, the cost of this examination is not included.

Finally, in the case of a lesion identified on ultrasound, a cerebral scan is added to the toxoplasmosis examination at birth.

3. Follow-up of children

The assumptions outlined below were made according to the protocol described in Annex-Part 4, describing the management of children with CT in Paris, Lyon, and Marseille centers. Other approaches are possible but could not be sufficiently documented in this work to be taken into account.

3.1 No suspicion of child infection (negative neonatal screening, unrecognized seroconversion, and normal birth examinations)

No specific monitoring is performed. Toxoplasmosis may be discovered at a later time, especially when a retinochoroiditis occurs.

3.2 In the case of suspected congenital toxoplasmosis

a. Confirmed CT (prenatal and / or neonatal diagnosis)

Asymptomatic and symptomatic children receive the same treatment during the first 12 months of life. Adiazine / Malocide or Fansidar® combined with folinic acid is administered continuously for a minimum of one year and a maximum of one year and a half. An average duration of 1 year and 3 months was applied in the tree.

The dosage for Malocide® is 1mg / kg / day (one time-a-day), that of Adiazine® is 100 mg / kg / day (two times-a-day). The dosage of Fansidar® is 1/4 tablet / 5kg each 10 days. Finally, folinic acid is prescribed at a dose of 50 mg per week. The mean weight of a child at 3 months is 5.75 kg, 7.75 kg at 6 months, 8.75 kg at 9 months and 9.75 kg at 12 months. The average dose received during the first year is thus ¼ tablet of Fansidar® every 10 days. The average weight between 12 and 18 months being 10.5 kg, the dose of Fansidar® then becomes ½ tablet every 10 days. Nine tablets are therefore counted for the first year and five for the second year, i.e. a total of 14 tablets and therefore 5 boxes. Before treatment initiation, a first blood count is performed, followed by a second one 15 days later, and then monthly; overall, 13 blood counts are performed during the first

year of life and 3 during the second year. Monthly proteinuria should only be performed if a combination of malocide and adiazine is prescribed (12 controls during the first year, 3 during the second year). Moreover, it is necessary to plan a clinical assessment (pediatric consultation), a specific ocular examination (including fundus exam) and a serology (B40) every 3 months, i.e. 4 controls during the 1st year. A semi-annual supervision is usually planned during the second year of life: two controls are thus included. The monitoring is then annual. The horizon adopted for the tree is 15 years; thus 13 toxo-related examinations are accounted for between 2 and 15 years, and overall the total for the whole follow-up corresponds to 19 toxo examinations.

b. Suspected congenital toxoplasmosis without confirmation by antenatal examinations or at birth

If the child is not infected, a toxo serology (B40) is performed at an average of 1 month of life, and then every 3 months from the 3rd month (6, 9, 12 months) until complete negativity is confirmed on two consecutive serologies. This occurs usually before the age of one year. A deadline of 6 months was applied. Negative status is therefore considered confirmed on average during the 9th month of life (after 3 checks).

If the child has been infected, without a clear confirmation by the different systematic exams realized, the above surveillance scheme is applied. As soon as the serological follow-up indicates asymptomatic CT (increase of IgG, appearance of specific IgM / IgA), a treatment similar to that proposed when the CT is confirmed is introduced. The diagnosis of CT is confirmed on average at 3 months of life (data from Toxo-Ly). Fansidar® is prescribed at a dose of ¼ tablet up to 1 year (requiring 7 tablets) and then ½ tablet for the next 6 months (9 tablets). This represents a total of 17 tablets and therefore 6 boxes.

c. Symptoms occurring during the 15-year follow-up

In the case of CT, the first ocular lesions are generally discovered before the age of 5 years, although later onset lesions may also be observed. The assumption is made that these lesions are discovered on average at about 2 ½ years of age, the child weighing on average 13.5 kg at that time. We considered that treatment is advised only in the case of active lesion, which was observed in 16.5% of cases in the Toxo-Ly cohort. Fansidar® is taken for 3 months in this case at the rate of ½ tablet every 10 days, 5 tablets for the whole treatment (2 boxes). Treatment monitoring will require a blood count before treatment, then a second one 15 days later and finally a monthly monitoring for a total of 4 blood counts. An ocular examination is planned at the end of the treatment to check the healing.

These lesions may recur or other lesions may appear and eventually reach the second eye. These recurrences may occur in 29% of cases (Toxo-Ly data). A new 3-month sequence of Fansidar® is then prescribed. Recurrence may occur within 3 months to 12 years after the initial lesion (median: 3 years). Very few children have repeated recurrent lesions; thus we did not account for this type of event. Based on these data, we assumed ocular lesion recurrence to occur on average at 5.5 years (weight of child at this date: 19.5 kg). Treatment with Fansidar® is then prescribed at the dose of 1 tablet every 10 days, i.e. 9 tablets or 3 boxes for this sequence of treatment. The supervision is identical to that described above for the initial episode.
